# What Drives the US and Peruvian HIV Epidemics in Men Who Have Sex with Men (MSM)?

**DOI:** 10.1371/journal.pone.0050522

**Published:** 2012-11-29

**Authors:** Steven M. Goodreau, Nicole B. Carnegie, Eric Vittinghoff, Javier R. Lama, Jorge Sanchez, Beatriz Grinsztejn, Beryl A. Koblin, Kenneth H. Mayer, Susan P. Buchbinder

**Affiliations:** 1 Department of Anthropology, University of Washington, Seattle, Washington, United States of America; 2 Department of Biostatistics, Harvard School of Public Health, Boston, Massachusetts, United States of America; 3 Department of Epidemiology and Biostatistics, University of California San Francisco, San Francisco, California, United States of America; 4 Asociación Civil Impacta Salud y Educación, Lima, Peru; 5 Asociación Civil Impacta Salud y Educación, Lima, Peru; 6 Instituto de Pesquisa Clinica Evandro Chagas, Fundação Oswaldo Cruz, Rio de Janeiro, Brazil; 7 Laboratory of Infectious Disease Prevention, New York Blood Center, New York City, New York, United States of America; 8 The Fenway Institute, Fenway Health, Boston, Massachusetts, United States of America; 9 Bridge HIV, San Francisco Department of Public Health, San Francisco, California, United States of America; Rollins School of Public Health, Emory University, United States of America

## Abstract

In this work, we estimate the proportions of transmissions occurring in main vs. casual partnerships, and by the sexual role, infection stage, and testing and treatment history of the infected partner, for men who have sex with men (MSM) in the US and Peru. We use dynamic, stochastic models based in exponential random graph models (ERGMs), obtaining inputs from multiple large-scale MSM surveys. Parallel main partnership and casual sexual networks are simulated. Each man is characterized by age, race, circumcision status, sexual role behavior, and propensity for unprotected anal intercourse (UAI); his history is modeled from entry into the adult population, with potential transitions including HIV infection, detection, treatment, AIDS diagnosis, and death. We implemented two model variants differing in assumptions about acute infectiousness, and assessed sensitivity to other key inputs. Our two models suggested that only 4–5% (Model 1) or 22–29% (Model 2) of HIV transmission results from contacts with acute-stage partners; the plurality (80–81% and 49%, respectively) stem from chronic-stage partners and the remainder (14–16% and 27–35%, respectively) from AIDS-stage partners. Similar proportions of infections stem from partners whose infection is undiagnosed (24–31%), diagnosed but untreated (36–46%), and currently being treated (30–36%). Roughly one-third of infections (32–39%) occur within main partnerships. Results by country were qualitatively similar, despite key behavioral differences; one exception was that transmission from the receptive to insertive partner appears more important in Peru (34%) than the US (21%). The broad balance in transmission contexts suggests that education about risk, careful assessment, pre-exposure prophylaxis, more frequent testing, earlier treatment, and risk-reduction, disclosure, and adherence counseling may all contribute substantially to reducing the HIV incidence among MSM in the US and Peru.

## Introduction

Three decades on, the HIV epidemic in the United States and other highly developed nations remains concentrated among men who have sex with men (MSM), with over half of new infections occurring in this community [1]. The same is true throughout Latin America [2]. In the US, HIV incidence is rising among young MSM, especially Blacks [3]. Incidence trends for Latin American MSM are less well characterized, although prevalence remains high in this community throughout the region [4].

Recent advances in pre-exposure prophylaxis [5], testing [6], antiretroviral treatment-as-prevention [7] and circumcision [8], [9], [10] have raised hopes for HIV prevention, including among MSM. While none alone is likely to stop the epidemic, larger reductions in incidence may be achieved by prevention packages, tailored to individual risks and preferences, that combine some or all of these new interventions with new and existing counseling approaches that support adherence, more frequent HIV testing, and continued sexual risk reduction.

Optimizing prevention packages will require a better understanding of HIV transmission in these populations. Recent work has estimated the proportion of new infections among developed-world MSM that occur within main partnerships [11], [12], [13], [14], during acute infection [15], [16], [17], [18], [19], [20], [21], [22], [23], [24], and by the sexual role [13], testing history, and treatment status of the infected partner [22], [25]. These studies use methods ranging from cross-sectional modeling to phylogenetics, start from varying assumptions, and typically address only one or two of these questions. Similar work on MSM transmission dynamics in developing world settings is lacking.

In this paper we jointly estimate these key epidemiological measures among urban MSM populations in the US and Peru. Both countries have epidemics concentrated among MSM [2], and, despite population differences in socioeconomic status, health care access, and sexual behaviors, HIV prevalence among urban MSM in each country appears to be roughly similar [26], [27]. This work represents the baseline modeling results for the Prevention Umbrella for MSM in the Americas (PUMA) project, which aims to identify more effective ways of combining and targeting HIV interventions for MSM throughout the Western Hemisphere.

## Methods

### Overview

We use dynamic, stochastic, network models, one for the US and one for Peru, parameterized using multiple large-scale behavioral and demographic surveys of MSM, including NHBS [26], [28], Peru’s Sentinel Surveillance [29], HPTN-036 [30], and the baseline surveys of HPTN-039 [31] and Explore [32], [33]. All are focused on large urban centers, and most have some definition of high risk for their inclusion criteria, so our models reflect these populations. The models simulate parallel networks for main partnerships and casual sexual contacts, both implemented using exponential random graph models (ERGMs) in the R package suite *statnet* [34], with some extensions. The main/casual distinction parallels that from source studies, which generally define a main partnership in terms of emotional commitment. Additional model components are also programmed in R. Model features and inputs were determined in consultation with a team of HIV researchers and community advisory board members. Unlike compartmental models, network models are not easily presented through flow diagrams; instead, we provide an analogous summary of model structure in Table 1. Additional detail, including parameter values, is provided in the Supporting Information.

**Table 1:** Model features.

Agent attributes. Each man (agent) in the model possesses the following attributes:		Network models. The probabilities governingrelations between pairs:		Transitions. The following changes can happen to men:	
Age		Main partnerships evolve as a function of:		Entrance into population	
	Race (US only)		Age of men	Aging	
	Circumcision status		Race of men (US only)	Infection	
	Sexual role preference		# of partnerships men are already in	Change in viral load	
	Propensity for casual UAI	UAI occurs w/in main partnerships as function of:		Progression through disease stages	
	Infection status		Disclosure and diagnosis status of men	Testing	
	Diagnosis status		Disease stage of men	Treatment initiation	
	Viral load (for HIV+ men)		Men’s sexual roles	Death from AIDS	
	Stage of infection (for HIV+ men)	Casual UAI occurs as a function of:		Death from other causes	
	Treatment status (for HIV+ men)		Age of men		Sexual retirement
	Treatment adherence and suppression		Race of men (US only)		
	Testing propensity		Men’s propensity for casual UAI		
	Duration since sexual debut		Men’s disclosure and diagnosis status		
	Duration since last negative test		Disease stage of men		
	Duration since infection		Men’s sexual roles		
	Duration since positive diagnosis				

### Overall Population

Our models work by simulating the day-by-day history of a population of MSM, beginning with 10,000 men. Individuals vary according to fixed characteristics including race, circumcision status, sexual role, and propensity for UAI in casual sexual contact; sexual role is modeled as varying continuously from exclusively insertive to exclusively receptive. Men enter the population at age 18 and are followed until age 65 or death, whichever comes first. The assumed entry rate results in slow population growth in the presence of a stable HIV epidemic. Non-AIDS-related mortality rates are based on published life tables; in men who never receive treatment, disease progression to AIDS occurs 10 years after HIV infection, and death from AIDS occurs 2 years later; progression is slower and survival greater for men on ART, and varies by magnitude of adherence and viral suppression (see below). Models were begun with 19% prevalence [26], run through a burn-in period to eliminate the transient effects of seeding the infection arbitrarily, and then compared to observed HIV incidence and prevalence in these populations. We discuss the burn-in in more depth in the online technical supplement, and its relationship to our outcomes. Each scenario is modeled 10 times, for 25 years.

### Main Partnerships

Day by day, we simulate the formation and subsequent dissolution of main partnerships, the occurrence of unprotected anal intercourse (UAI) within them, and HIV transmission if one partner is infected. Under the ERGM, main partnership formation reflects each potential partner’s sexual role, race, age, and current partnership status. Partnerships between men with incompatible sexual roles (two strictly insertive or receptive men) are disallowed. After formation, the daily risk of main partnership dissolution is assumed constant, so partnership duration follows a geometric distribution. If either partner is infected, daily UAI probability varies by whether the infection has been diagnosed and disclosed. UAI rates are further reduced once either man develops clinical AIDS. Sexual role may vary across UAI episodes, according to the sexual roles of both partners.

### Casual Sexual Contacts

In parallel, we simulate UAI in casual contacts and resulting HIV transmission. Specific contacts reflect the sexual role, age, race, and HIV serostatus of each partner, as well as his propensity for UAI in casual contacts. As with main partnerships, contacts between men with incompatible sexual roles are disallowed, and only UAI risk is considered.

### HIV Transmission

When a UAI contact occurs between serodiscordant partners, per-contact HIV transmission risk is made to depend on the role of the uninfected partner, and if he is insertive, whether he is circumcised. In the US model, circumcision prevalence varies by race, and declines over time, while for Peru prevalence is constant. Per-contact transmission risk is also made to depend on the time since infection of the infected partner as well as his treatment status, mediated by viral load, as described below.

Transmission probabilities are not as well documented for MSM as for heterosexuals; the one serodiscordant couple study of transmission by time since infection focused on heterosexual transmission [35], as have all three studies of circumcision [8], [9], [10]. Our initial approach (Model 1) focused exclusively on viral load differences as the source of acute hyperinfectiousness. However, new research published during the course of this project suggests that acute infection viral strains may be more infectious even after accounting for viral load [36], [37]. We thus implemented a second variant (Model 2) that considered these additional potential sources. We explore both models for both countries.

In *Model 1*, we based infection rates on viral load and per-contact risk in the absence of treatment, modeled with parametric curves [38], [39], [40]. These estimates did not take into account stage of infection independent of viral load. Viral load rises and falls over 40 days post-infection; chronic infection lasts for a subsequent ten years in the absence of treatment, followed by two years of rising viral load during late-stage infection; then death. Per-contact risk ratios by sexual role and circumcision status of an uninfected insertive partner were determined by estimates for anal [41] and vaginal sex [8], respectively.

In *Model 2,* we used infection stage and per-contact risk in the absence of treatment, without explicit consideration of viral load. The acute stage is assumed to last two months, with infectiousness rising in the first month and falling in the second; subsequent changes in per-contact risk follow the patterns from Model 1, except for the slope of infectiousness during late-stage. We derived risk ratios for the *average* risk in each stage from estimates of relative per-contact risk by stage for UAI among heterosexuals [35]; infectiousness slopes during acute and late stages were fit to yield these average stage-specific relative risks. Risk ratios by role and circumcision status were carried over from Model 1. Given uncertainty in absolute risk in this framework, per-contact risks were calibrated to yield HIV prevalence for the stable epidemic equal to Model 1.

**Figure 1:**
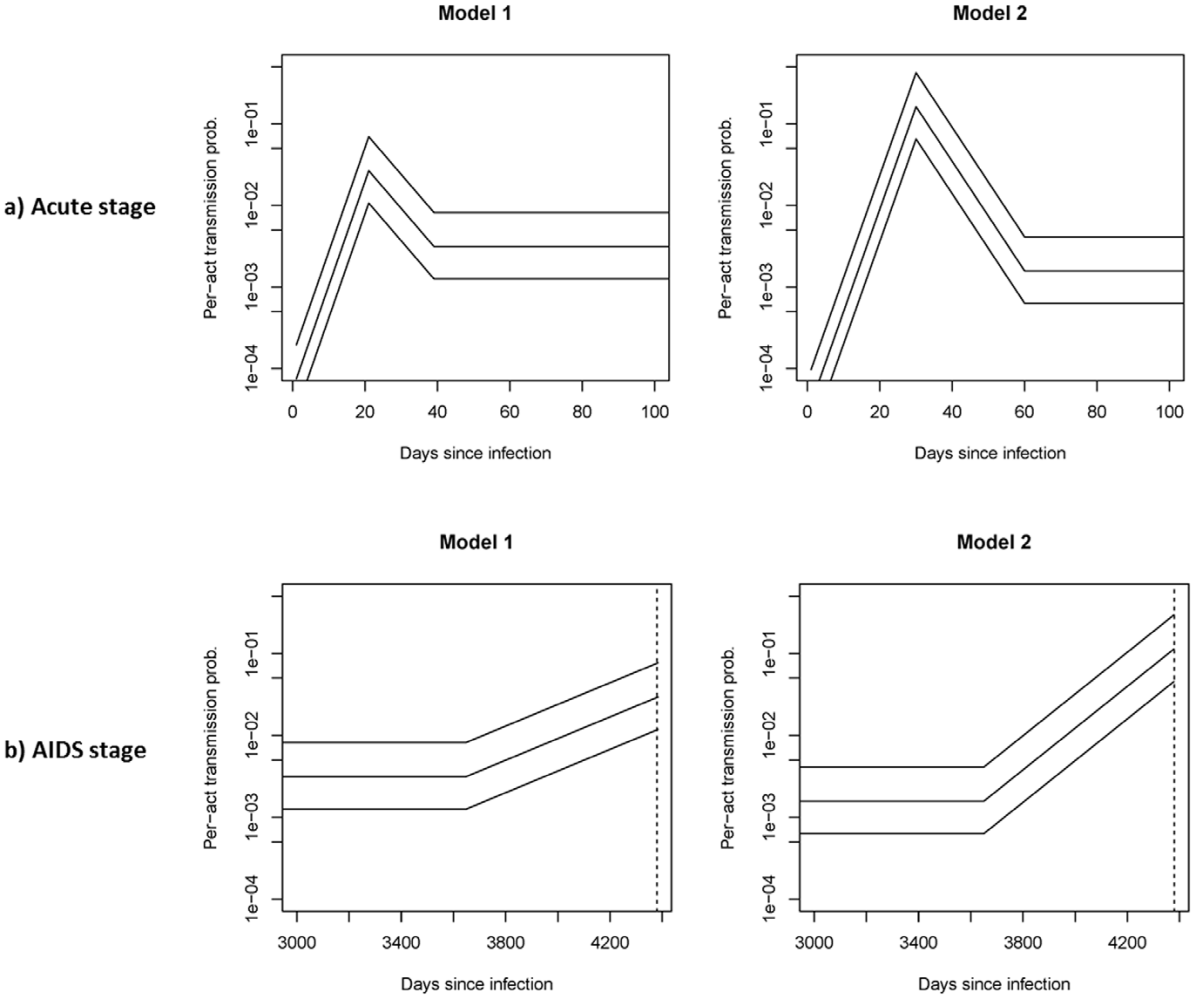
Per-act infectivity by time since infection, during acute stage (**Figure 1a**) and AIDS state (**Figure 1b**). For more information on the derivation, see the online technical supplement.

The infectiousness curve implied by each model in the absence of treatment is summarized by Figure 1a (acute stage) and Figure 1b (AIDS stage).

### Testing, Treatment, and Infectiousness

We simulate HIV testing, and, after an infection has been diagnosed, when treatment begins. In both models, infectiousness on treatment is mediated by viral load.

Men become eligible for treatment initiation when their expected CD4+ count, modeled as a function of time since infection [42], drops below country-specific thresholds, which are also race-specific for the US. Thresholds are determined by observed CD4+ counts at initiation, and are driven by a variety of factors such as testing frequencies and access to health care. Explicitly modeling a wide range of patterns of treatment initiation, levels of adherence, cessation, and potential re-initiation was beyond the scope of the model, since the data needed to do so were limited (particularly in terms of survival for men with arbitrarily complex treatment histories). Instead, we reduce this complexity down to three representative trajectories: a proportion of randomly selected men in each country never initiate treatment; among those who do, some achieve partial and some full viral suppression, depending on adherence. Parameters are set to achieve the prevalence of three states in cross-sectional data: the proportion of men who are not on treatment; on treatment and partially suppressed; and on treatment and fully suppressed. Rates of progression to AIDS and death, as well as infectiousness, vary across these groups. In particular, per-contact risks are motivated by published estimates of mean viral load [43] for each group, in combination with the relationship of viral load to infectiousness from Model 1. With disease progression, viral load and thus infectiousness increase again until death [40]. Results suggest a 93% reduction for full suppression as compared to untreated men, qualitatively similar to the 96% recently reported [7].

### Sensitivity Analyses

Models 1 and 2 vary in their treatment of infectiousness early in infection. To obtain estimates of uncertainty due to key model inputs, we conducted sensitivity analyses varying three particularly uncertain and/or influential inputs: duration of main partnerships; relative frequency of UAI in main partnerships and casual contacts; and HIV testing frequency.

## Results

### Prevalence and Incidence

In Model 1, prevalence at steady state across repeated simulations averages 28% for the US and 29% for Peru, and mean annual incidence is 1.8% and 1.9%, respectively. The US prevalence estimate is slightly higher than the 25% found in most cities in the first MSM round of NHBS [44], an expected pattern given that most source datasets excluded MSM with no HIV risk. For Peru, there is less clear documentation of recent incidence and prevalence. Reports of HIV prevalence for Lima in the early 2000s were just above 20%, and increasing by 6% year over year [27]; if that trend has continued, prevalence should be ∼30% in recent years. Other Peruvian cities have lower prevalence, but our model mainly reflects inputs for Lima, home to 73% of the country’s diagnoses [45]; source data here also exclude men with no HIV risk.

### Infection Stage, Diagnosis and Treatment

**Figure 2:**
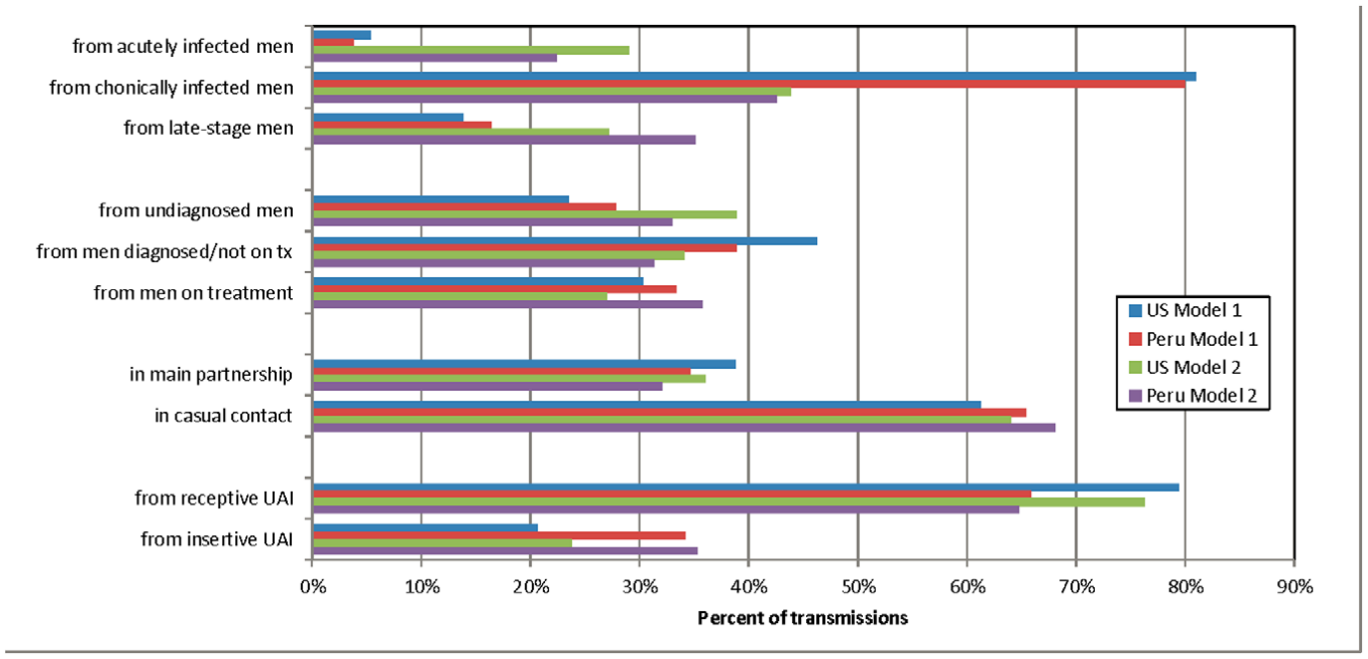
Distribution of transmission events by key variables. Bars represent the means across ten stochastic simulations over 25 years. The variance of these estimates across runs was less than 2 percentage points for all measures, so only means are shown. For year-on-year variation, see the Figure 3 and the online technical supplement.

Model 1– in which infectiousness is mediated by viral load–predicts only a small role for acute infection (5% in the US and 4% in Peru) and a dominant role for chronic infection (81% and 80%, respectively; Figure 2). In contrast, Model 2–which uses a stage-based model for viral load in the absence of treatment– estimates a more equal balance, but still a plurality for chronic (29%, 44%, 27% for acute/chronic/late in the US; 22%, 43%, 35% in Peru). The distribution of transmissions by detection and treatment also varies by model; nevertheless, in all cases transmissions are roughly balanced across the undiagnosed, untreated, and treated groups.

### Proportion of HIV Transmission Occurring within Main Partnerships

Model 1 consistently estimates that UAI within main partnerships is the source of ∼39% of infections in the US and ∼35% in Peru, despite constituting 52% and 48% of UAI acts, respectively. Results for Model 2 are slightly lower.

### Sexual Role

Transmissions by sexual role showed the greatest international difference. Model 1 estimated that in the US, only 21% of transmission was from the receptive to the insertive partner, compared to 34% for Peru; Model 2 is qualitatively similar. This can be partly attributed to the lower prevalence of circumcision–assumed to lower UIAI risk by 60%–in Peru, in combination with greater role exclusivity; in the latter context, transmission to more-or-less exclusively insertive men must play a bigger role in sustaining chains of infection.

### Variation in Outcomes

**Figure 3:**
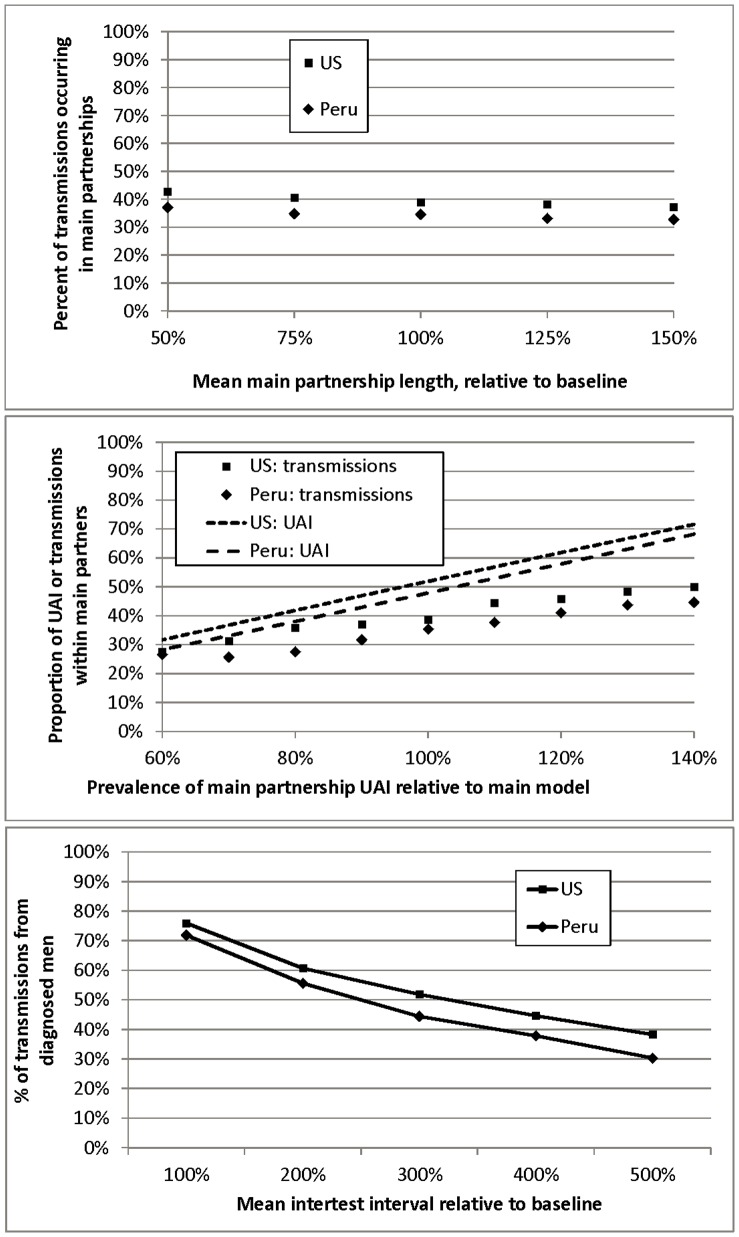
Range of variation from year to year for outcome metrics. Each boxplot covers the values for a given outcome metric measured for each of the 25 years in a single run. We show US Model 1 here as demonstration; comparable plots for the other three country/model combinations are in the online technical supplement.

Our metrics on transmission types varied little across runs within the same scenario, never ranging over more than 2 percentage points. This is due to both our large population size and to the long time period (25 years) over which we modeled them. Variation in metrics was of course higher over the short term, and this short-term variation can be of interest. Figure 3 shows the distribution for each outcome metric when measured for each of the 25 years separately, for the US Model 1. Analogous plots for the other country/model combinations are in the online technical supplement. Each shows a qualitatively similar pattern: for our population, these metrics see most of their variation lie with a roughly ten percentage-point range. It is important to remember that the magnitude of this range is still a function of our population size (∼10,000, with roughly 150 new infections per year). Smaller populations would see more variation; those that are larger, with sexual partnerships forming throughout the population, would see less.

### Sensitivity Analyses

**Figure 4:**
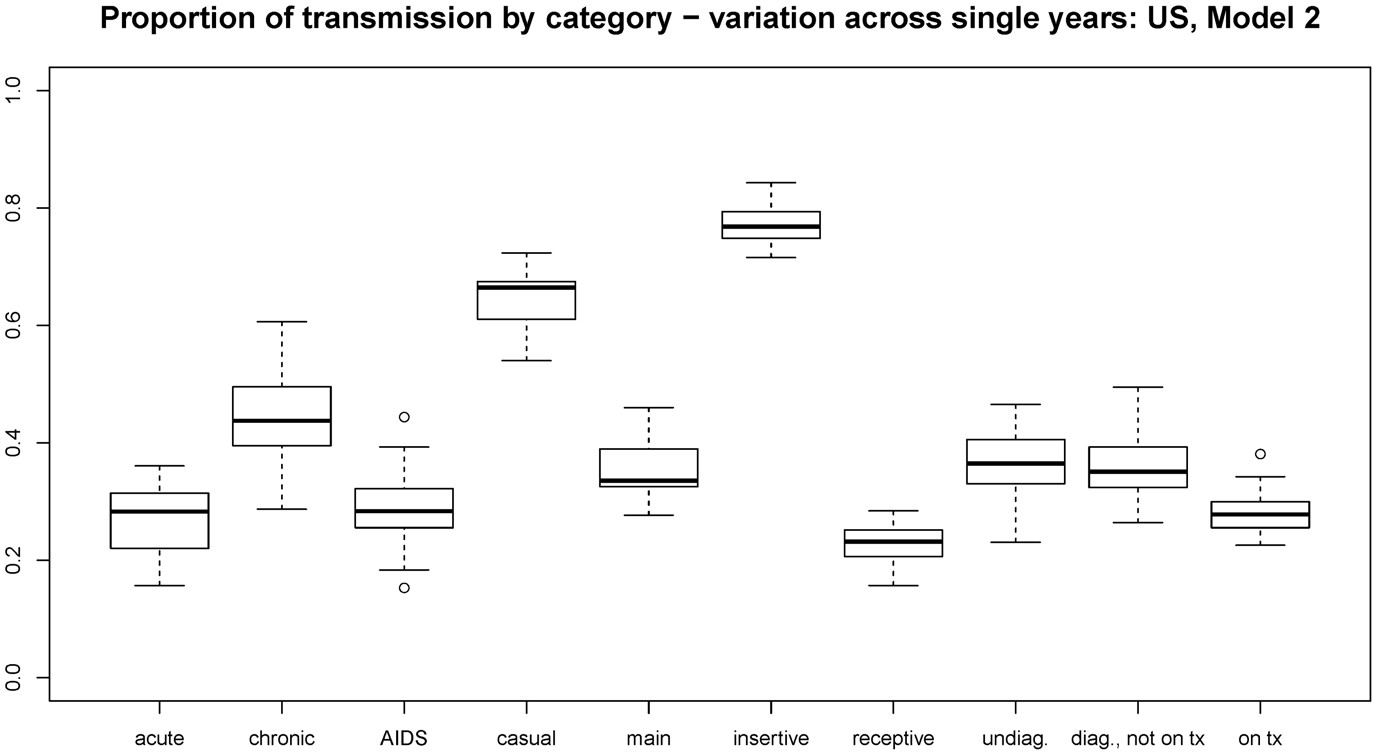
Sensitivity analyses. a) Changes in main partnership duration relative to base model (100%). b) Changes in main and casual UAI relative to base model (100%). c) Changes in testing frequency relative to base model (100%).

Analyses were conducted to assess sensitivity to three particularly uncertain model inputs. We present these for Model 1 in Figure 4. Average main partnership duration is hard to estimate, with a strongly right-skewed distribution, censoring, and recall bias. We varied this input, holding the prevalence of main partnerships constant (Figure 4a). Results were only mildly sensitive to partnership length.

Given the divergence between our estimates of the proportion of transmissions occurring in main partnerships and other published estimates, we explored the impact of different assumptions about the relative frequency of UAI in main partnerships versus casual contacts (Figure 4b). Specifically, we increased the frequency in main partnerships by 10–40%, simultaneously reducing its frequency in casual contacts by the same percentage. As expected, the proportion of transmissions occurring in main partnerships correlates positively with the relative frequency of UAI in these partnerships. However, it remains much lower than the proportion of all UAI *contacts* occurring in these partnerships, reflecting the fact that transmission can only occur once in any partnership. Moreover, the estimated proportion of main-partner transmissions never exceeds 50%, even with very large increases in the relative UAI frequency; this reflects the need for casual contacts to sustain chains of infection.

Data on the proportion of men reporting testing in the last year, used in our models, generally suggest higher testing frequency than estimates based on self-reported infection status among men testing positive [26], [44], [46]. Even with testing at half our estimated frequency (Figure 4c), men with diagnosed infection generate a majority of transmissions. At lower testing rates, this proportion necessarily drops, but reaches a lower bound; in addition, HIV prevalence in these models increases to less realistic levels.

## Discussion

Our models are the first to comprehensively and consistently address several questions about HIV transmission, including the proportions of transmissions occurring during acute as well as undiagnosed, untreated, and partially suppressed infection, within main partnerships, and to insertive partners in anal intercourse. Our results help clarify these questions for MSM in sites on two continents, with implications for prioritizing prevention efforts and designing combination prevention interventions, including the PUMA package currently in development.

Although variation in infectiousness soon after HIV acquisition is not well understood for MSM, leading us to develop two variants of our models, nonetheless both suggested that a considerably smaller percentage of transmissions occur during acute infection than some recent estimates based on phylogenetic clusters [15], [18], [24], [47]. Newer work has critiqued the statistical methods used in some of those papers, and developed their own estimates based on similar data types [16], [17]; these, as well as other modeling estimates [19], [20], [23], lie closer to our more conservative estimate of 4–5%. Our less conservative estimates of 22% in Peru and 29% in the US are more similar to one modeling estimate for Australia [22]. Recent work [36], [37] suggesting that additional factors beyond just viral load induce high transmissibility during acute infection would argue for the less conservative estimates, although further clarification on acute infectiousness for MSM is urgently needed. The lower estimate for Peru may reflect lower circumcision prevalence, later treatment initiation, and less suppression due to lower adherence among the treated, all of which make post-acute transmission more likely. However, all of our estimates suggest that strategies to increase testing for men regardless of stage of infection may be more fruitful than some phylogenetic work seems to imply, and that efforts focused narrowly on the more difficult task of identifying acute-stage men are not as crucial.

Moreover, our models suggest that similar proportions of transmissions stem from undiagnosed, diagnosed but untreated, and inadequately-treated men in both countries. This argues that earlier treatment initiation and strategies to maintain retention in care and adherence to treatment must be linked to testing, and all should be included in comprehensive prevention packages. These data are consistent with recent findings from two different studies that <30% of HIV-infected Americans are virologically suppressed [48], [49].

None of the six CDC “evidence-based” interventions for MSM address transmission within stable partnerships, which comprise 35–39% of all infections in our results, comparable to other recent estimates of 33% [11] and 26% [12]. While two recent developed-world estimates (86% [14] and 68% [13]) were considerably higher, the first of these defined stable partnerships more broadly and focused on young men, who switched steady partners frequently; the second is based on a simpler model that does not explicitly account for dynamic network structure, and uses a different estimate of relative UAI frequency in stable partnerships vs. casual contacts (Sullivan, personal communication). Despite the variability of these estimates, they are consistent in suggesting that substantial HIV transmission occurs in main partnerships in both the US and Peru, and thus support development and evaluation of behavioral interventions for MSM couples. Definitions for main partnerships were similar in source studies (generally reflecting emotional commitment), but the nature of such partnerships may differ considerably by country, and needs to be better understood.

Less is known about the proportion of HIV transmission from receptive to insertive anal sex partners. We estimated that 21% of transmissions are in this direction, similar to an earlier estimate of 28% [13], for US MSM; the slight difference may reflect the fact that our model explicitly requires the numbers of receptive and insertive acts to be equal. We found no estimates of transmissions by role for Peru. Our estimate of 33% is higher than for the US, despite the apparent belief among some MSM in Lima that the insertive partner is at very low risk. This misconception leads to another: specifically, that exclusively insertive men are highly unlikely to be HIV infected and therefore pose no risk to their HIV negative receptive partners. Our findings suggest that messages addressing these misconceptions should figure prominently in risk reduction counseling for MSM with *any* sexual role, especially in the context of greater role exclusivity. Another implication is that promotion of circumcision might be a useful intervention among nearly or exclusively insertive MSM, at least in South America, where circumcision is relatively uncommon and seems protective among highly-insertive men [50]. However, cost and limited uptake could limit the effectiveness of this approach, especially since exclusively insertive men are disproportionately heterosexually-identified, and thus likely to not be open about their sexual activities with other men [51]. Counseling messages should emphasize that although receptive anal sex carries the highest risk, a substantial proportion of infections is also caused by insertive anal sex.

As with all models, our approach has numerous limitations. Some parameters are based on one data source, especially for Peru, and inputs are generally subject to bias and sampling error. Stage-specific estimates of per-act transmission were only available for heterosexuals; similar MSM measures are urgently needed. Although our model was more detailed than most, modeling always requires simplification, in part because data for more complicated models are commonly lacking. For example, we did not model temporal variability in treatment effectiveness, possibly leading to some underestimation of risk in main partnerships, where undetected treatment failure would be risky if partners relied on full suppression rather than condoms. Also, while our study represents an advance on earlier models in explicitly modeling main and casual partnerships separately, we did not explicitly model partnerships involving repeated contacts with partners not perceived as “main,” but rather treated these as separate casual contacts. This could understate the importance of long-term relational concurrency, and provides no explicit guidance for counseling MSM about these relationships. It could also affect overall incidence in our model, and underestimate the proportion of infections in main partnerships by allowing someone to transmit to multiple casual partners in the model who are in reality the same person. In addition, we did not model contacts with females, a population at potentially substantial secondary risk in each setting. However, given the relatively low HIV prevalence among heterosexual women in both countries, female-to-male transmissions are unlikely to play any substantive risk for MSM in the Americas, as opposed to settings of generalized epidemics, e.g. southern Africa. We did not explicitly model male sex work or transgender identity, two aspects of MSM life of particular importance in Peru; however, men involved in either were included in source studies and thus contribute to model parameters. Finally, our data were limited to two countries–the US and Peru. Generalizing either model to other countries in the Americas should only be done with caution, as there remains much work to do in determining whether key inputs are similar for MSM in other settings.

In a pre-planned extension of this work, we will model the potential effects of various interventions in MSM, including pre-exposure prophylaxis (PrEP), which could dramatically change the prevention landscape for MSM. We will also assess the maximal predicted impact of voluntary medical male circumcision, which is likely to be incorporated into many prevention packages for heterosexuals in Africa, but whose impact in MSM, even in populations with substantial role segregation, is less clear. Using data from these models, we are building a combination prevention package for MSM in North and South America. The PUMA package is likely to include PrEP (with strategies to improve medication adherence), home HIV testing with online and telephone support and linkage to care, couples-based interventions, and more explicit risk reduction counseling information. We hope that through the combination of these prevention strategies for HIV uninfected persons and improved treatment coverage and adherence in HIV infected persons, we can halt the growing HIV epidemic in MSM throughout the Americas.

## Supporting Information

File S1 **Online technical supplement.**
(DOCX)
