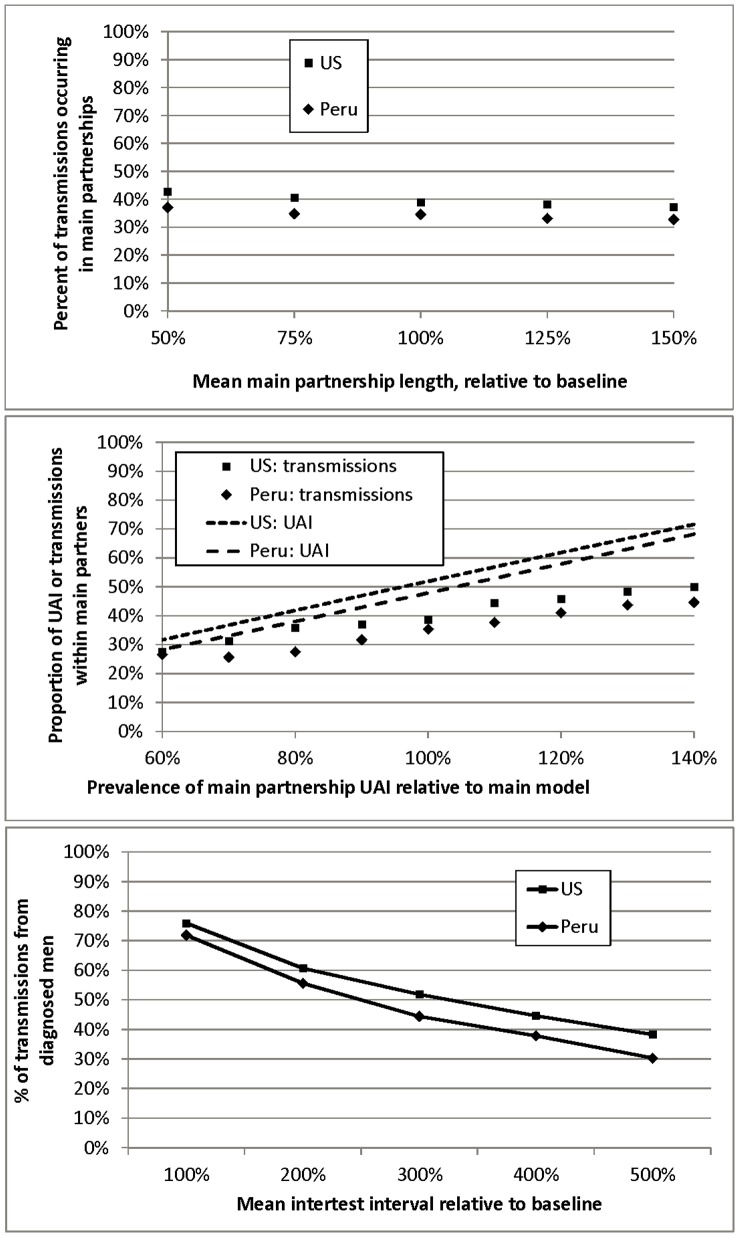# Correction: What Drives the US and Peruvian HIV Epidemics in Men Who Have Sex with Men (MSM)?

**DOI:** 10.1371/annotation/9a6a0c8e-2d01-4f36-9ab8-f9fdfce6497b

**Published:** 2013-07-17

**Authors:** Steven M. Goodreau, Nicole B. Carnegie, Eric Vittinghoff, Javier R. Lama, Jorge Sanchez, Beatriz Grinsztejn, Beryl A. Koblin, Kenneth H. Mayer, Susan P. Buchbinder

Figures 3 and 4 are currently in reverse order. Their legends are in the correct order.

Figure 3. Range of variation from year to year for outcome metrics.

Each boxplot covers the values for a given outcome metric measured for each of the 25 years in a single run. We show US Model 1 here as demonstration; comparable plots for the other three country/model combinations are in the online technical supplement.

doi:10.1371/journal.pone.0050522.g003

Please see figure 3 at the following link:

**Figure:**
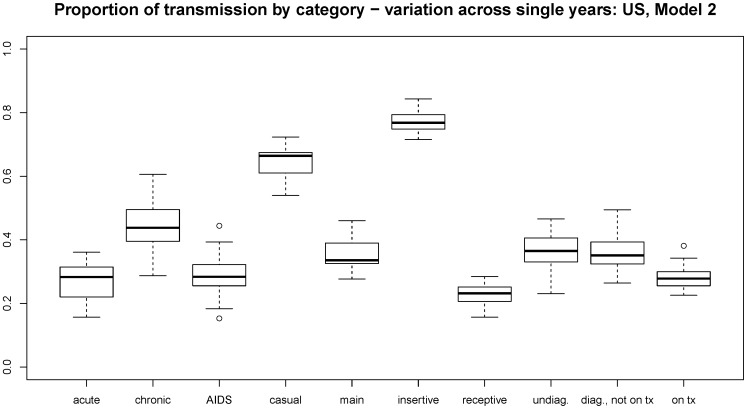


Figure 4. Sensitivity analyses.

a) Changes in main partnership duration relative to base model (100%). b) Changes in main and casual UAI relative to base model (100%). c) Changes in testing frequency relative to base model (100%).

doi:10.1371/journal.pone.0050522.g004

Please see Figure 4 at the following link:

**Figure:**