# Supplementary material for: What Drives the US and Peruvian HIV Epidemics in Men Who Have Sex with Men (MSM)?
Source: PLoS One. 2012 Nov 29;7(11):e50522. doi: 10.1371/journal.pone.0050522 (PMC3510067; doi:10.1371/journal.pone.0050522)
Supplement: File S1 — Online technical supplement. (DOCX) [file pone.0050522.s001.docx]

**ONLINE TECHNICAL SUPPLEMENT**

**DATA SOURCES AND PARAMETERS**

We list data sources and derived parameter values in the three tables below; following these, we provide greater detail about the model structure itself, the derivation process for each parameter, and the assumptions that drove them. Within these tables, a cited source indicates that the estimates were obtained from published data, whereas a named study indicates that the estimate was derived from new analysis of original data from an existing study or personal communication with study staff. These studies include Explore [[1](#_ENREF_1),[2](#_ENREF_2)], HPTN-036 [[3](#_ENREF_3)], HPTN-039 [[4](#_ENREF_4)], the National HIV Behavioral Surveillance System 2008 MSM data from San Francisco (NHBS-08-SF) [[5](#_ENREF_5)], the National HIV Behavioral Surveillance System 2003-5 data from New York (NHBS-05-NY) [[6](#_ENREF_6)] [[7](#_ENREF_7)], Peru 2008 Sentinel Surveillance (study unpublished; see [[8](#_ENREF_8)] for description of previous rounds of the sentinel surveillance), Project T [[9](#_ENREF_9)], the Step Study [[10](#_ENREF_10),[11](#_ENREF_11)], and the HIV Outpatient Study (HOPS) [[12](#_ENREF_12)].

Abbreviations used in the tables include:

- Diagnosis status: DP = diagnosed positive; NU = negative or undiagnosed; UNK = unknown.
- Studies: NHBS = National HIV Behavioral Surveillance System; HPTN = HIV Prevention Trials Network; HOPS = HIV Outpatient Study.
- Acts: UAI = unprotected anal intercourse; URAI = unprotected receptive anal intercourse; UIAI = unprotected insertive anal intercourse
- Race/ethnicity: B= Black, L = Latino, O = Other.

A note on race and ethnicity: for the US, we considered three categories, based on their relevance to HIV epidemiology among MSM. These were Black, Latino, and Other. Our categories were hierarchical in this order; for example, a man who identifies as both Black and Latino is categorized as Black. In all cases where we had access to original data, we categorized men as such. In some secondary sources, we were restricted to using their categorical divisions. In most cases, we could simply aggregate data into our categories (e.g. summing their White, Asian, Native American and Other categories into our Other category) In other cases this was not possible; the most common was when sources included Black Latinos with Latinos rather than Blacks. We assume the effects of using the data with slightly different definitions to be small.

TABLE S1: Parameters with region-specific values

| Model parameter(s) | Source(s) and value(s), US | Source(s) and value(s), Peru |
| --- | --- | --- |
| Initial prevalence | 19%  NHBS-08, all cities [[5](#_ENREF_5)].  Note that although this is set at the beginning of the burn-in period, it achieves a new equilibrium independent of this initial value before the end of the burn-in, as a result of natural epidemic dynamics. | 19%  consistency with the US  Note that although this is set at the beginning of the burn-in period, it achieves a new equilibrium independent of this initial value before the end of the burn-in, as a result of natural epidemic dynamics. |
| Age-specific non-AIDS mortality rates | All–cause mortality: National Vital Statistics Reports Vol. 56, No. 9 [[13](#_ENREF_13)].  AIDS mortality (to subtract from all-cause in order to get non-AIDS mortality): National Vital Statistics Reports Vol. 58, No. 8 [[14](#_ENREF_14)]. | Source: WHO life tables, data for Peru males for 2008 [[15](#_ENREF_15)]. |
| Baseline daily probability of casual UAI | Explore  Placed in five quintiles: 0.0020, 0.0129, 0.0292, 0.0610, 0.2246 | HPTN-036  Placed in five quintiles: 0.0005, 0.0076, 0.0231, 0.0479, 0.2097 |
| Age mixing within casual contacts | NHBS-08-SF  Mean difference in the absolute value of the square root of the ages of casual contacts = 0.73. | Peru 2008 Sentinel Surveillance  Mean difference in the absolute value of the square root of the ages of casual contacts = 0.77 |
| Diagnosis status mixing (serosorting) in casual contacts | NHBS-08-SF.  The conditional log-odds of a contact increase by 0.78 if two men have the same diagnosis status. | HPTN-039  The conditional log-odds of a contact increase by 0.24 if two men have the same diagnosis status. |
| Momentary (cross-sectional) degree distribution for main partnerships | Project T, Explore, NHBS-08-SF, HPTN-039  0 main partnerships = 60.0% of men  1 main partnership = 38.3% of men  2 main partnerships = 1.7% of men | HPTN-036  0 main partnerships = 57.5% of men  1 main partnership = 40.9% of men  2 main partnerships = 1.6% of men |

| Main partnership mean duration | NHBS-08-SF  1120 days | Peru 2008 Sentinel Surveillance; HPTN-036  809 days |
| --- | --- | --- |
| Age mixing within main partnerships | NHBS-08-SF Mean difference in the absolute value of the square root of the ages of main partners = 0.61. | Peru 2008 Sentinel Surveillance  Mean difference in the absolute value of the square root of the ages of casual contacts = 0.76 |
| Prevalence of disclosure in main partnerships | Explore, NHBS-08-SF, HPTN-039  90% of main partners disclose their status to each other as they know it (DP, NU). The remaining 10% do not. | HPTN-036  40% of main partners disclose their status to each other as they know it (DP, NU). The remaining 60% do not. |
| Daily probability of UAI within an existing main partnership, by disclosure and diagnosis status | Explore  undisclosed 0.114  disclosed + - 0.108  disclosed - - 0.167 | HPTN-039  undisclosed 0.076  disclosed + - 0.057  disclosed - - 0.144 |
| Age distribution | Initially set as uniform from 18-65; the 100-year burn-in period ensures that the age distribution settles down to what is appropriate for the given arrival and departure rates as well as the HIV transmission and mortality experience. Model exploration begins after the end of burn-in. | Initially set as uniform from 18-65; the 100-year burn-in period ensures that the age distribution settles down to what is appropriate for the given arrival and departure rates as well as the HIV transmission and mortality experience. Model exploration begins after the end of burn-in. |
| Circumcision status for pop. coming of age prior to the present | The STEP study  Black=85%, Latino=50%, Other=91% | Peru 2008 Sentinel Surveillance  0.05 |
| Circumcision status for pop. coming of age after the present | National Hospital Discharge Survey (http://www.cdc.gov/nchs/nhds.htm)  Black=70%, Latino=50%, Other=60% | Peru 2008 Sentinel Surveillance  0.05 |

| Prevalence of role exclusivity | Explore  Total role exclusivity by race:  Black: 26.8%, Latino 17.7%, Other 14.6%  Two forms of exclusivity (insertive and receptive) assumed to be equal within race | Peru 2008 Sentinel surveillance.  Exclusively insertive: 22.7%  Exclusively receptive: 27.2% |
| --- | --- | --- |
| Daily probability of HIV test | Helms et al.2009 [[16](#_ENREF_16)] and others  0.002849 | Peru 2008 Sentinel Surveillance  0.0016 |
| Treatment initiation | Swindells et al. 2002 [[17](#_ENREF_17)], Lyles et al. 2000 [[18](#_ENREF_18)]  After both of the following criteria are met:   1. One has tested positive. 2. One has been infected for 4.1 years (Black men), 5.0 years (Latino men) or 3.6 years (Other men). | Moreira et al. 2011 [[19](#_ENREF_19)]  After both of the following criteria are met:   1. One has tested positive. 2. One has been infected for 3.9 years. |
| Percent of positive men never receiving treatment throughout their entire lives | 15%. Expert consultation (see notes). | 15%. Expert consultation (see notes). |
| % of men going on treatment who achieve full suppression | HOPS, Chu et al. 2010 [[20](#_ENREF_20)], Weintrob et al. [[21](#_ENREF_21)]  62.7% (Black), 68.8% (Latino), 74.9% (Other) | Ministry of Health of Peru [[22](#_ENREF_22)], expert consultation  65% |
| Proportion of individual UAI contacts that involve intra-event role versatility | NHBS-08-SF  22% of main UAI contacts between two versatile men;  32% of casual UAI contacts between two versatile men. | HPTN-036  24% of main UAI contacts between two versatile men;  14% of casual UAI contacts between two versatile men. |

TABLE S2: Parameters unique to the US

| Race composition | NHBS-2005 (all cities) [[6](#_ENREF_6)]  17.5% Black, 27.0% Latino, 55.5% Other |
| --- | --- |
| Race mixing in main partnerships | NHBS-05-NY and NHBS-08-SF. The following matrices represent the proportion of partnerships for the row group that are with the column group. These numbers are adjusted from the data to be consistent with the racial composition of the model population. Note that under random mixing, each group would have 17.5% of their ties with Blacks, 27.0% with Latinos, and 55.5% with Others:   \|  \| Black \| Latino \| Other \| Total \| \| --- \| --- \| --- \| --- \| --- \| \| Black \| 54.9% \| 19.4% \| 25.6% \| 100.0% \| \| Latino \| 12.6% \| 38.0% \| 49.4% \| 100.0% \| \| Other \| 8.1% \| 24.1% \| 67.9% \| 100.0% \| \|  \|  \|  \|  \|  \| |
| Race mixing in casual contacts | NHBS-05-NY and NHBS-08-SF. The following matrices represent the proportion of partnerships for the row group that are with the column group. These numbers are adjusted from the data to be consistent with the racial composition of the model population. Note that under random mixing, each group would have 17.5% of their ties with Blacks, 27.0% with Latinos, and 55.5% with Others:   \|  \| Black \| Latino \| Other \| Total \| \| --- \| --- \| --- \| --- \| --- \| \| Black \| 52.1% \| 24.5% \| 23.3% \| 100.0% \| \| Latino \| 15.9% \| 34.4% \| 49.7% \| 100.0% \| \| Other \| 7.4% \| 24.2% \| 68.4% \| 100.0% \| \|  \|  \|  \|  \|  \| |

TABLE S3: Parameters shared across regions

| Initial population size | 10,000 men |
| --- | --- |
| Arrivals | Poisson with mean of 0.685 per day to ensure very slight growth in the face of a generalized HIV epidemic |
| HIV test window period | US Food and Drug Administration Talk Paper No. T01-42.  22 days |
| Reduction in UAI once AIDS stage is reached | Wawer et al. [[23](#_ENREF_23)]  40% |
| Model 1: Time until peak of acute viremia | Little et al. [[24](#_ENREF_24)]  21 days |
| Model 1: Peak viremia (in log10 copies per ml) | Little et al. [[24](#_ENREF_24)]  6.886 |
| Model 1: Time from peak viremia until set point | Little et al. [[24](#_ENREF_24)]  19 days |
| Model 2: Duration of acute infection | Laynaert et al. [[25](#_ENREF_25)]  60 days |
| Model 2: Mean infectivity during acute infection relative to chronic | Laynaert et al. [[25](#_ENREF_25)]  16.8 |
| Models 1 and 2: Set point (in log10 copies per ml) | Little et al. [[24](#_ENREF_24)]  4.5 |
| Total time from infection until AIDS-related viral increase | Buchbinder et al. [[26](#_ENREF_26)]  10 years |
| Time from onset of AIDS-related viral increase until death | San Francisco City Clinic Cohort data ([[26](#_ENREF_26)] and unpublished data)  2 years |
| Model 1: Slope of viral load during AIDS | .003425 log10 copies per ml per day  This implies a viral load of 7.0 at death |
| Model 2: Mean infectivity during AIDS infection relative to chronic | Laynaert et al. [[25](#_ENREF_25)]  16.8 |
| Viral load at full suppression | 1.5.  Must be below 1.7 (log10 of 50) by definition |
| Mean viral load for those partially suppressed (in log10 copies per ml) | Chu et al. 2010 [[20](#_ENREF_20)]  3.5 |
| Time from initiation of treatment until suppression (full or partial) | Chu et al. 2010 [[20](#_ENREF_20)]  90 days |
| Time until partial suppression escape | Chu et al. 2010 [[20](#_ENREF_20)]  Thirteen years from infection |
| Partial escape slope | 0.003425 log10 copies per ml per day  chosen for consistency with untreated slope |
| Model 1: Probability of transmission, by act and circumcision status of negative partner, by log10 viral load (vl) of positive partner | Circumcized, UIAI with positive (2.23 *x* 10^-5^)(2.45^vl^)  Uncircumcized, UIAI with positive (5.57 *x* 10^-5^)(2.45^vl^)  URAI with known positive (1.45 *x* 10^-4^)(2.45^vl^) |

**SECTION S2. MODEL STRUCTURE**

The overall process consists of (1) generating an initial population; (2) estimating main and casual network models on that population; (3) running the population through a burn-in period; (4) simulating epidemics. The basic components of both the burn-in and the simulation phases, repeated at each time step, are: (a) main partner network formation and dissolution; (b) determination of UAI within main partner network; (c) generation of a casual UAI network; (d) transmission within UAI events; (e) update of other attributes (e.g. testing status, viral load); (f) vital dynamics (entrances, exits, aging). We implement both network estimation and simulation using *statnet* (*http://www.statnetproject.org*).

**2. Main partnership model.**  The log-odds of main partnership formation on a given day between any two men in the population were modeled as a linear function of the following factors:

- the race combination of the two men
- the absolute difference in square-root of age between the two
- whether this would be a second ongoing main partnership for either or both men

We disallowed new main partnerships involving two exclusively insertive or two exclusively receptive men, or any man who was already in two ongoing main partnerships. Existing relationships had a constant daily hazard of dissolution, implying a geometric survival time.

Expected values for a given network for race mixing matrices, mean absolute age differences, momentary degree distributions (i.e. the distribution of the number of partnerships men are in at any snapshot in time), and relational durations were derived from data as explained in the tables above. Because of the presence of the momentary degree terms, the log-odds of each relationship is conditionally dependent on the status of other relationships in the network. This recursive dependence makes it impossible to estimate the parameters using traditional likelihood methods, and also makes it impossible to estimate any parameter in the model in isolation of the others. The network modeling framework known as exponential random graph models (ERGMs) exists precisely to solve this problem for cross-sectional models [[27](#_ENREF_27),[28](#_ENREF_28)]. The general ERGM form specifies the probability of observing any set of relationships *y* on a set of *n* actors as:

$$P\left(Y=y\left| n\text{ actors}\right.\right)=\frac{\exp\left(\sum_{k=1}^{K} \theta_{k}z_{k}\left(y\right)\right)}{c}$$

where *z_k_* is a vector of *k* different network statistics, proposed to affect the probability of relational formation; *θ_k_* represents the coefficients on those statistics (as in more familiar generalized linear models); and *c* represents the quantity in the numerator summed over all possible networks on the same actor set, in order to produce a proper probability distribution. The normalizing constant renders the expression impossible to evaluate using standard techniques, requiring simulation-based algorithms such as MCMC in order to obtain the maximum likelihood estimates for *θ_k_* for a given set of network statistics *z_k_* [[29](#_ENREF_29)]. A recently developed extension known as separable temporal ERGMs (or STERGMs; Krivitsky and Handcock 2010) extends this framework to dynamic networks like the one here. We employ this framework to jointly estimate all of the parameters governing our main partnership model; in the process we rely on the efficient approximation method for STERGMs derived by Carnegie et al [[30](#_ENREF_30)], and dynamic extensions to the R *network* package [[31](#_ENREF_31),[32](#_ENREF_32)].

Parameter estimates were then used to drive the main partner network simulation module of the larger epidemic simulation discussed below. For both the main partnership and casual contact networks, we use the offset method of Krivitsky et al. [[33](#_ENREF_33)] during the simulation phase, to adjust for the changing size and composition of the network through time. This approach ensures that the underlying mean number of partnerships per person remains in place as the population grows or shrinks, and allows the race and age mixing to adjust to any changes to the underlying population composition appropriately. At all stages of the project, simulated main partnership networks were checked to ensure that they indeed retained the expected cross-sectional structure and relational durations throughout the simulations.

**3. Casual contact model.** The casual UAI model is a cross-sectional one, with contacts formed each day independently of the previous. Thus, it is governed by a single equation rather than two. It also has additional terms in it beyond those in the main partnership model, since it is modeling UAI specifically. (The determination of UAI within existing main partnerships occurs outside the ERGM framework in our model, and involves these same additional predictors; see below).

The casual UAI model assumed that the log-odds of a contact occurring between any two men on any given day were a function of:

- each man’s underlying propensity for casual UAI (“activity class”; explained below)
- the race combination of the two men
- the absolute difference in square-root of age between the two
- the HIV diagnosis status of each man

This model was estimated using traditional cross-sectional ERGMs, and parameter estimates were used to drive the casual contact module of the epidemic simulations. As with main partnerships, we used Krivitsky’s offset method during simulation, and checked all output for consistency with the target network structure.

**4. Model parameters**

**4.a. Casual activity class**. It is inaccurate to assume that men are homogeneous with regard to their propensity to engage in casual UAI; the variance in the distribution of number of acts over the last year in all of our data sources makes this clear. That said, we have little information on the continuity in men's UAI risk from year to year over their lives, and the degree to which men transition through levels of risk. In order to maintain the high variance in the casual UAI behavior distribution, we partition men into five activity classes, each of which has a mean underlying daily risk of casual UAI equal to the mean in the corresponding quintile of the observed data. Unfortunately, surveys rarely to never ask about UAI in terms of rates (acts of UAI with all partners per unit time, for instance). So we must estimate these numbers by interpolating, often imperfectly, from sets of related questions.

For US: We used baseline data from Explore. We used partner-specific questions on UAI during last sex along with partner’s relationship type and status to determine population-level probabilities of UAI given any sex by partner type and serostatus. We then combined these with individual reports on numbers of partners in the last six months by serostatus, presence or absence of steady partner in the last six months, and reports on numbers of sex acts with partners by status in the last six months. Collectively, this gave us estimates for numbers of casual UAI acts in the previous six months, which we converted into daily probabilities.

For Peru: We used data from Peru-036. We combined questions on total number of male partners in the previous three months, numbers of casual partners among the three most recent (assuming all others were casual, for those who had more than three), UAI during most recent sex act with each partner, and whether or not sex had occurred multiple times with each partner. In the end, we had four extreme outliers whose reports implied multiple casual sex partners per day, and whose presence in the model yielded an equilibrium prevalence of over 50%; they did not report sex work, and the patterns of values suggested key punch errors. Removing them generated an epidemic that much more closely resembled that of Lima. As a form of confirmation, we also analyzed the Peru Sentinel Surveillance data. These were less informative, since the study only asked about date of first and last sex within relationships. Nevertheless, we used questions about the length of time since the last act of casual UAI for men, combined with counts of partners, to yield estimates. This approach yielded an overall rate of casual UAI remarkably similar (to the third decimal place) as that from Peru-036 with the outliers removed.

**Race mixing.** For race mixing, data were made available from the second round of NHBS from San Francisco, as well as the first round of NHBS for New York City. The ERGM approach automatically adjusts for the fact that the racial composition of each city is different. In both cases, we found qualitatively similar results, despite the fact that two cities stand on opposite sides of the average NHBS city (both have about the same percentage Latino as the average city, but SF is more Other and less Black, while NY is more Black and less Other). The six types of main partnerships, from most likely to least, were the same (BB, LL, OO, OL, LB, OB, where B=black; L=Latino, O= other). The coefficients for the two cities were quite consistent as well. BB and OB stood out for being the most distinct; LL and OO were qualitatively similar, as were OL and LB. For casual contacts, SF had the six combinations in the same order, while NY saw LL and OO switch positions and OL and LB switch positions (the most similar combinations to begin with). Given the qualitative similarities between the two cities, we averaged across cities to obtain the log-odds of each relational combination for each relational type.

**Age mixing.** For both main and casual, we use the difference in the *square root* of men’s ages, following Krivitsky et al. [[33](#_ENREF_33)] The use of the square root of age provides closer fit to the observed matrix than does age directly; this statistic also has several nice properties, including the fact that the same absolute difference in age becomes less important with older ages. For the US: Taken from NHBS-SF. We divided both respondents and their reported main partners into eight age categories (18-20, 21-25, 26-30, 31-35, 36-40, 41-45, 46-50, >50). We then assumed that within each group men were distributed uniformly by age (>50 was held to 50-55 for symmetry). We then calculated the square root of each man’s age, and determined the absolute value of the difference in these for each partnership, and averaged this. For Peru: Taken from Peru Sentinel Surveillance 2008. We used the same strategy, although for this dataset the available age categories were 18-20, 21-25, 26-30, 31-35, 36-40, 40+.

**Diagnosis status mixing for casuals.** For the US: we use reports of respondent diagnosis status (DP, NU), along with their reports of their casual partners’ status (DP, NU, UNK). We reassigned UNK men to DP or NU using the assumptions that 19% of men were true positives (CDC 2008), and 90% of those were diagnosed. Note that the latter figure is much higher than the rate of diagnosis found in NHBS, but it corresponds to the rough proportion who would be diagnosed given data on testing frequencies from the same study. See the main paper for more on this topic. These yielded estimates for DP contacts with NU and NU contacts with DP. We assumed the midpoint for our point estimate, and used the resulting prevalence of the three contact types in our ERGM model. For Peru, we only had reports from DP men, who reported most of their contacts as UNK. We re-distributed UNK to DP and NU using the same algorithm as for the US, and then used the observed prevalence for the three partner types in our ERGM model.

**Partnership durations.** Our approach to modeling relational dissolution implies that relational duration will follow a geometric distribution. A key feature of this distribution means that the expectation for the mean age of ongoing relationships at a randomly selected point in time is equal to the expectation for the mean duration of relationships after completion over a long period of time. This property allows us to use data on the existing age of ongoing relationships from cross-sectional surveys to parameterize dissolution. For the US, we used NHBS-08-SF. This survey asked for the duration of sexual relationship with the most recent partner and partnership type. Two other data sources were able to provide estimates of different types. The first is the Gay Couples’ Study (Hoff, personal communication), a prospective longitudinal study of male couples in San Francisco; for this, the proportion of men lost to follow-up at each round due to relational dissolution (taken as a weighted average across all rounds) provides an estimate of daily probability of relational dissolution. The second is Explore, which also asked about relational duration with most recent partners, although only categorized crudely (less than 1 month, less than 6 months, etc.) The former might be expected to oversample highly stable couples, while the latter would be expected to oversample men with unstable relationships given the high-risk entry criteria. Indeed, the NHBS-SF estimate is between the other two and near their mean, with the Hoff estimate of relational duration longest and the Explore estimate lowest..

. For Peru, we had two data sources. The 2008 sentinel surveillance yielded a mean ongoing main partner relational age of 728.5, which corresponds to a daily dissolution probability of 0.001373. HPTN-036 yielded a mean ongoing main partner relational age of 908.4, which corresponds to a daily dissolution probability of 0.001101. The mean of these two probability estimates is 0.001237, which corresponds to a duration of 809 days.

**Steady partner disclosure.** We assumed that when men disclose, they do so as they know their status. For the US, we assumed that 90% of couples have disclosed their statuses as they know them to be, and 10% have not. (True disclosure is of course a dynamic process, not a static one, so we only aim to reproduce its prevalence in the cross-section accurately). We derived our estimate using responses to questions about partner's HIV status, which typically include options of positive, negative, or I Don't Know; we coded the last as undisclosed and the former two as disclosed. Questions are typically asked about the status of the partner at the most recent sex act. Three different studies provided information on this, which were all in approximate agreement. Measured this way, disclosure was 85.4% in Explore, 95.0% in NHBS-II-SF, and either 87.3% or 93.0% in HPTN-039, according to two different ways of analyzing the data (by act or by partner). We took the approximate center of all these points, 90%. For Peru, we use the same metric from the one study that we have which measures it (HPTN-036).

**Steady partner daily probability of UAI.** This depends on the disclosure and diagnosis status of the couple, and also on whether either has progressed to AIDS. Couples who disclose are assumed to do so with the truth as they know (DP vs. NU). For the US, rates of UAI for undisclosed couples, disclosed seroconcordant negative, and disclosed serodiscordant were taken from Explore; for Peru, we derive the same parameters from HPTN-039.

**Momentary degree distribution for main partnerships.** For the US, none of the main studies (Explore, NHBS-SF, HPTN039) asked this question directly. However, they all allowed one to put an upper and lower bound on the number, where upper = % with any main partner over a recent time window (typically 6 months or a year), and lower = living with partner or similar stringent measure. This yields prevalence of a main partner as:

Explore: 24.4% to 50.2%

NHBS-SF: 34.1% to 43.0%

HPTN039: 26.4% to 54.2%

Project T asked this question directly, and found that 40% of men in a main partnership on the day of the interview (Liu, personal communication). As this number lies in the center of all three of these distributions, we used it as our prevalence for having at least one main partner.

We then wished to estimate the percent of men in more than one main partnership. Again, no study asked this directly. However, it is important to not simply forbid it in our model (which would underestimate the importance of main partners as men themselves define them), nor to leave it unparameterized (which would result in far too many men having multiple main partnerships). Explore asked a highly complex set of questions in two separate modules about partner-specific dates of most recent sex, relational duration (categorized as “less than one week”, “less than one month”, etc.), and partnership type (but not present relational status), as well as prevalence of main partnerships at any point in the last six months. From these we could determine upper and lower bounds as 2.8% and 0.6% of the population in two main partnerships at any given time. Lacking any other information, we simply took the average (1.7%). Given the low prevalence of 2+ concurrent main partnerships, and the lack of additional data, we imposed a constraint in our model of no more than two main partnerships at a given time.

For Peru, data on the momentary degree distribution for main partnerships was much more straightforward, as the question was asked directly in HPTN-036. Note that the estimates for the US (which required a variety of assumptions) match those for Peru (which were measured directly) quite closely.

**Treatment trajectories.** The real world of treatment is complex; men can go on or off treatment, switch among many different regimens, and have different levels of adherence and resistance to the treatments they are on. The set of available treatments is also constantly changing. We cannot hope to model all of this complexity; instead, we consider three different treatment states: not on treatment (NT), on treatment and fully suppressed (FS) and on treatment and partially suppressed (PS). There are not sufficient data to be able to determine the patterns and rates at which individual men switch among these three states over the course of their infection, particularly since the treatment landscape is constantly evolving. Instead, we model individual men as being on one of three types of trajectories (never treatment, treatment with full suppression, treatment with partial suppression) such that our model matches the prevalence of full suppression and treatment-naïve death in the population as a whole. For suppression in the US, we have two highly concordant estimates for the population as a whole. In HOPS, 74% of men who have consistently been on HAART for at least six months show full suppression. For Chu et al. the figure is 75% [[20](#_ENREF_20)]. Weintrob et al. were the only published estimates found to break the data out by race [[21](#_ENREF_21)]. They also showed exactly 75% suppression for European-American men, but only 62.7% for African-American men. These were the only two populations they considered. We use their European-American figure for our Other population, and their African-American figure for our Black population. We arbitrarily assume that Latinos are the mid-point of the two, for lack of any data. For Peru, we had one estimate of 76% suppression after rollout [[22](#_ENREF_22)]. However, this occurred in an initial, and heavily monitored rollout context, and consultation with Peruvian HIV experts suggested this was too optimistic an estimate for overall suppression. We reduced the figure to 65% in consultation with those experts, placing the figure in line with our estimates for Blacks and Latinos in the US. For proportion of HIV+ MSM who are treatment-naïve at AIDS death, we were unable to find any recent estimate for either the US or Peru. The only published estimate found used data that is now over a decade old, and not limited to MSM [[34](#_ENREF_34)]. Thus, we settled on a value of 15% for both the US and Peru, through a process of expert consultation with the PUMA research team, which includes scientists from the US, Peru and Brazil. The estimate for proportion dying treatment-naïve can be used directly for the proportion of men who enter that trajectory at seroconversion; however, the observed prevalence of full vs. partial suppression must be weighted using the expected durations in each state (which differ due to life expectancy differences) in order to determine the proportion of new seroconverters who enter each trajectory. We assumed a duration with partial suppression of 15 years (net of deaths from other causes). Full suppression was assumed to be effective indefinitely (also net of deaths from other causes). Simulations were checked to ensure that the prevalence of each trajectory among the HIV+ population were correct. Log10 viral load was modeled as 4.5 for chronic infection in the absence of treatment; 3.5 for partially suppressed men, and 1.5 for fully suppressed men, each derived as described in Table S3.

**Reduction in UAI once AIDS stage is reached.** We could find no data for MSM specifically and used numbers derived from Wawer et al. [[23](#_ENREF_23)] for heterosexuals in Uganda.

**Treatment initiation.** For the US, treatment start time varies by race. Swindells et al. [[17](#_ENREF_17)] found that median CD4+ count at initiation was 220 (Latinos), 318 (Blacks), and 372 (Whites). We use the “Whites” figure for our Other category. Note that although these figures are a few years old now, much more recent data that did not distinguish by race [[19](#_ENREF_19)] found a comparable value of 366 overall for MSM in Baltimore, a population that is largely a mixture of Black and White. Since we do not model CD4+ count explicitly, we must convert these numbers into expected times since infection. Lyles et al. (2000) provides an expression for expected CD4+ count since first positive visit, the latter of which averages three months after infection. Including those three months yields an average treatment initiation time by race (in years after infection) of 5.0 (Latinos), 4.1 (Blacks), 3.6 (Whites). Although these numbers are old, they are the most recent that we could find. For Peru, the only estimates for MSM that we were able to find for Peru [[22](#_ENREF_22)] represented documentation of the initial country-wide rollout of expanded services, so that most new enrollees were far advanced. We found more representative data for Rio de Janeiro. There, Moreira et al. developed a model-based estimate of mean CD4+ of 342 for MSM at first presentation [[19](#_ENREF_19)]. Using the functional form of Lyles yields and estimate of 3.9 years after infection. For both the US and Peru, men must also be tested in addition to reaching the appropriate time since infection in order to begin treatment. Treatment initiation is deterministic, once both of these criteria are met.

**Model 1 approach to transmission.** We wish to obtain estimates for probability of transmission from HIV-positive to HIV-negative man, for arbitrary viral load in the HIV-positive man, differentiated by sex act (unprotected insertive anal intercourse = UIAI, unprotected receptive anal intercourse = URAI), and by circumcision status when the HIV-negative man is insertive. We begin with Vittinghoff et al. [[35](#_ENREF_35)], who estimated per-act transmission probabilities to a negative man of:

URAI with known positive 0.82%

URAI with known positive or unknown 0.27%

UIAI with known positive or unknown 0.06%

The estimates do not include UIAI with a known positive. We determine a rough point estimate for this by calculating the ratio of UIAI with positive/unknown to URAI with positive/unknown to (=.06/.27 = .22) and then applying this to URAI with positive. This yields an estimate for UIAI with positive = .22 * .82% = .18%. Of course, these numbers still do not consider circumcision or viral load.

To consider circumcision, we assume the same *proportional* reduction as in the Rakai study of Uganda heterosexual sex, where circumcision reduced the probability of acquisition by 40% during insertive penile-vaginal sex [[36](#_ENREF_36)]. We assumed that the Vittinghoff estimate for UIA was a weighted average across circumcised and uncircumcised men, with Vittinghoff’s population reflecting the overall circumcision prevalence of the adult male US population [[35](#_ENREF_35)]. This yields new UIAI estimates, along with our existing URAI estimate, of:

Circumcised, UIAI with positive 0.126%

Uncircumcised, UIAI with positive 0.314%

The estimate for URAI does not depend on circumcision status, and this remains at 0.82%. Next, we assume that most or all of the known positives that respondents reported contact with had chronic infection. This is because few men in acute infection would be known to be positive, and men in late-stage infection are less likely to be having sex. (We return to this point in the description of Model 2 below). We assume that treatment effects were not large given that the study data on which the paper was based predated the rise of HAART. Given this, we assumed that the above estimates could be used as approximate values for the probability of transmission for a chronically infected man. We then plugged these three values for v_0_ into the expression for transmission by viral load from Wilson et al. [[37](#_ENREF_37)], along with their value of β_0_=4.5 as baseline chronic viral load, to obtain three separate curves for infection probability by viral load. This expression is:

β_1_ = 2.45^log10(v1/v0)^ β_0_

which yields probabilities of transmission by viral load v_1_ of:

Circumcised, UIAI with positive 0.00126 * 2.45^[log10(v1)-4.5]^

Uncircumcised, UIAI with positive 0.00314 * 2.45^[log10(v1)-4.5]^

URAI with known positive 0.00820 * 2.45^[log10(v1)-4.5]^

As heuristics, these formulas correspond to the following transmission probabilities:

|  | Circumcised,  UIAI | Uncircumcised,  UIAI | URAI |
| --- | --- | --- | --- |
| Chronically infected partner | 0.00126 | 0.00314 | 0.00820 |
| Partially suppressed partner | 0.00051 | 0.00128 | 0.00335 |
| Fully suppressed partner | 0.00009 | 0.00021 | 0.00056 |

The pattern of viral load during acute viremia is based primarily on the work of Little et al. (1999). For this period, we assume that viral load follows the following pattern, deterministically for each man:

Days 0-21: rises linearly from 0 to 6.886

Days 21-40: declines linearly from 6.886 to 4.5

We then assume a set point of 4.5, also from Little et al. (1999), which lasts until the onset of AIDS at ten years post-infection (Buchbinder et al. 1994):

Days 40-3650: equals 4.5

We then assume a linear increase in log viral load during AIDS up to a fatal viral load of 7.0, which lasts for two years (Buchbinder et al. 1994),

Days 3650- 4380: increases linearly from 4.5 to 7.0

Day 4380: death

The definition of full suppression in most studies is <50 copies of HIV/ml/. We assume that men who go on treatment and become fully suppressed achieve a log10 viral load of 1.5 (equal to 32 on the absolute scale). The actual value is fairly arbitrary, as it only affects the probability on onward transmission, which is virtually zero at these levels. Men are assumed to be able to maintain this status indefinitely, until they either die of other causes or reach 65 and depart from our population of interest. We remind that we are taking a schematic approach where these men represent the fully suppressed state of all men, with the cross-sectional prevalence (the only data available) matched

For the experience of men who are partially suppressed, we draw on Chu et al. [[20](#_ENREF_20)]. We model their partially suppressed viral load as 3.5 (taken from Figure 2a), with time from treatment initiation until suppression as three months. The same figure also suggests that for those with detectable viral load, escape towards no suppression begins in earnest about nine years after treatment initiation. We chose to model escape in terms of time since infection rather than treatment initiation, to avoid the perverse assumption that men who delayed treatment due to lower testing or other factors would have longer survival. Our men on average initiated treatment around year 4 of infection, so we modeled time until partial escape as 13 years from infection, regardless of when treatment was initiated. Once escape began, we modeled it as having the same slope as for those who were treatment naïve, for consistency and for lack of data.

Time from treatment initiation until suppression (whether full or partial) was modeled as 90 days, following the model of Chu et al. that suggested that just under half of men reached suppression at the 3 month mark [[20](#_ENREF_20)].

**Model 2 approach to transmission.** This approach focuses less explicitly on viral load during acute infection. Rather, it considers the relative transmissibility of HIV during each stage that was identified in Laynaert et al. for UAI [[25](#_ENREF_25)]. We use Laynaert rather than the more recent and widely-cited Rakai work, since only the former estimated time-varying risk of UAI, albeit for heterosexuals rather than for MSM. Specifically, Model 2 takes from Laynaert the following metrics: acute infection lasts 2 months (their best-fitting model), and per-act transmissibility averages 16.8 times higher during both acute and late-stage infection than chronic infection (derived by dividing 246.0/14.6 in Table 2). In this approach, we begin with the same chronic-stage transmissibility levels as in Model 1 for each of our three transmission types (uncircumcised UIAI, circumcised UIAI, receptive UIAI), but amplified the transmissibility during acute and late stages above that using these derived ratios. Per-act infectivity is not constant across acute and late stages. In the former it rises for one month and then falls for one month, following the same trajectory it would if log10 viral load were rising and falling linearly, but with the proper overall mean transmissibility. In the latter, it rises over time in the same way. For treatment, we modeled the impact of partial and full suppression on infectivity as in Model 1, although once viral escape happens, infectivity levels rise to the new late-stage values.

Model 2 initially resulted in incidence and prevalence figures that were considerably higher than Model 1. For the sake of comparability, we tried reducing the level of infectivity for all acts and circumcision combinations for chronically infected men in 10% increments (with absolute infectivity during acute and chronic infection and for all treatment statuses reducing in parallel). We found that an overall base infectivity of 60% of that used in Model 1 led to approximately the same (realistic) prevalence and incidence, and we adopted this set of absolute values for infectivity for Model 2. One potential justification for this comes from the method we used to derive chronic-stage infectivities from the mean values originally calculated by Vittinghoff et al. Our approach effectively assumed that all transmissions were occurring from chronically infected men, since few acute men would be diagnosed, and late-stage men have little sex. However, both of these groups likely contributed some transmissions to the observed data in Vittinghoff, even if it is fewer than chronics; and since their per-contact infectivity is so many times higher than chronically infected men, just a few such men could have pulled the mean infectivity far above the chronic infectivity level. Since we do not know the prevalence of such men among the source partners in the data used by Vittinghoff, the per-contact risk estimates in that study are really upper bounds on chronic infectivity; and values below that are all possible. Thus, we are using a chronic infectivity level within each model—each consistent with our incompletely observed data—that produces an epidemic similar to that observed empirically.

**Circumcision.** For the US, we used data from the STEP study (Susan Buchbinder, personal communication). Measures for the percentage of men circumcised (out of those who knew whether or not they were) yielded the estimates we used (Black=85%, Latino=50%, Other=91%) Note that HPTN-039 gave similar estimates (Black=90%; Latino=47%; Other=88%). For those coming of age later, we use lower rates, based on general trends from the last two decades. These numbers are only approximate, but we considered it important to capture the qualitative fact that circumcision prevalence is on the decline. We selected numbers reflecting average rates of circumcision by race over the last 20 years, rounded to the nearest 10%. The data were derived from the National Hospital Discharge Survey, as analyzed by CIRP (The Circumcision Information and Resource Pages; http://www.cirp.org/library/statistics/USA). For Peru, where circumcision rates are low and do not appear to be changing, we used the estimate from the Peru Sentinel Surveillance of 2006, which is a circumcision prevalence of 5%.

**Testing**. Helms et al. [[16](#_ENREF_16)] report a median inter-test interval of 243 days for MSM in four major metropolitan areas. Our model uses a constant daily probability of testing for simplicity; the value for this daily testing probability that corresponds to the median inter-test interval from Helms is 0.00285. This also corresponds to a *mean* test of about 351 days, or just short of a year. Given our (admittedly unrealistic) constant probability assumption, this implies that approximately 64.7% of men should report having tested in the previous year. This matches qualitatively what other large-scale population studies of MSM have found (one study [[38](#_ENREF_38)] reported that 92% of men had ever tested, and 64% had tested in the last year; another [[5](#_ENREF_5)] found that 58% of all men had tested in the last year, which rose to 64% when only considering men who had ever tested) *.* We did not include racial differences in testing due to the highly conflicting nature of the reports on this topic, and the lack of a significant difference in the data we had on hand.

For Peru, our one metric was the Peru Sentinel Surveillance data, which showed that only 44.7% of men had tested in the previous twelve months. The corresponding constant daily testing probability that yields this statistic is 0.00162. This corresponds to a mean inter-test interval of 617 days and a median of about 428 days.

**Role.** For the US, we use measures from Explore, the only one of our three core surveys that provided a clean measure for this. Men were asked about sexual behavior in the last six months, in each of up to eight surveys rounds. Men who had engaged in any anal intercourse in any survey were categorized according their reported role (exclusively insertive, exclusively receptive, or a mix) across all surveys. Proportions observed were:

|  | % exclusively insertive | % versatile | % exclusively receptive |
| --- | --- | --- | --- |
| Black | 19.6% | 73.2% | 7.2% |
| Latino | 10.9% | 82.3% | 6.8% |
| Other | 8.9% | 85.4% | 5.6% |

Those who reported versatile behavior were distributed fairly evenly throughout the distribution, with a small hump for each race at 50%.

The preponderance of exclusively insertive behavior over exclusively receptive behavior requires explanation, since in any given sex act there must be the same number of insertive and receptive partners. Possible explanations are:

1. The men who are versatile are receptive well over 50% of the time
2. This is a sample of negative men, and receptive men are disproportionately likely to become infected
3. There is some other form of sampling bias beyond serostatus
4. The data reflect desirability bias (with insertive sexual behavior being less stigmatized than receptive)

We are able to rule out Option 1 for our data by examining the versatile men’s reports. We have some indirect evidence for Option 2, in that an additional study (Project Enhance) indeed showed greater receptive behavior among HIV+ men (Kenneth Mayer, personal communication). Regardless of whether Option 2, 3, or 4 (or some combination of all of them) is true, it suggests that in the full population including both positives and negatives, the actual preponderance of exclusively insertive and receptive behavior should be closer to each other than they are here. For this reason, we kept the overall prevalence of role exclusivity by race as observed, but equalized the two types, yielding:

|  | % exclusively insertive | % versatile | % exclusively receptive |
| --- | --- | --- | --- |
| Black | 13.4% | 73.2% | 13.4% |
| Latino | 8.8% | 82.3% | 8.8% |
| Other | 7.3% | 85.4% | 7.3% |

For Peru, we use the Peru sentinel surveillance data, which asks directly about their role during anal intercourse over the previous five years. Responses were:

| % exclusively insertive | % versatile | % exclusively receptive |
| --- | --- | --- |
| 22.7% | 50.1% | 27.2% |

Here the ratio between exclusively receptive and exclusively insertive was closer (the larger was only 20% larger than the smaller), with a slight preponderance of receptive men. In this case, we could not rule out the possibility that the versatile men were insertive more than 50% of the time given our data. The bias towards receptive also means that Explanation 2 cannot hold here. Given this, we elected to keep the imbalance in our model, which means the versatile men in our simulation will indeed be slightly more insertive than receptive.

**Role selection within event.** When two versatile men have UAI (regardless of whether steady or casual), it is possible for either one or the other (or both) to be the insertive partner. The probability of intra-event versatility (that is, that both will be insertive and both receptive within a single sexual episode) varies by region and by steady vs. casual contact; these probabilities are derived from NHBS-08-SF (the US) and HPTN-036 (Peru) and are listed in Table 1. If there is not intra-event versatility, then one of the two men must be insertive and one receptive. Each versatile man possesses an insertivity value, drawn from the uniform distribution on (0,1); if these values for a pair of men are r1 and r2, then their respective probabilities of being the insertive partner are r1/(r1+r2) and r2/(r1+r2); roles are re-chosen with each individual act.

**BURN-IN.** The landscape underlying the HIV epidemic among MSM in both the US and Peru has shifted dramatically over time; e.g. through behavior change (especially early in the epidemic), the roll-out and expansion of testing, and continual improvements in treatment options. Although our primary interest is on the current state of the epidemic, that details of that current state is indeed dependent on the historical trajectory in a number of ways, some straightforward and some subtle. We are thus faced with taking one of a few possible avenues:

1. Attempt to model the entire history of the HIV epidemic from origin to present-day among our populations of interest, including major changes in behavior, testing and treatment
2. Begin our model at the introduction of infection into our population; use recent data on behavior, testing and treatment to parameterize the model for recent time periods, and arbitrarily change some behavioral or other parameters for the past to match various observed epidemic outcomes
3. Parameterize our model using recent data on behavior, testing and treatment, but begin the model at the initial seeding of the epidemic and run it until we reach realistic prevalence levels
4. Parameterize our model using recent data on behavior, testing and treatment, and begin the model at prevalence levels similar to today.

Approach 1 obviously comes closest to reality; however, given the complexity of our model, and the general shortage or inaccessibility of solid behavioral data for early decades, especially in Peru, this approach is unfeasible. Parallel efforts to unearth such data as there are and create a historical model for MSM are underway, but this is years from bearing fruit. Approach 2 is only moderately less feasible, and introduces some arbitrariness: since there are many behavioral parameters in our model, it is not clear which ones are appropriate to alter through time to achieve the desired trajectory. Approach 3 is infeasible for a different reason: the epidemic would have taken a very long time to achieve current prevalence given recent behaviors, and the computation time needed to run this full trajectory is excessive.

We thus adopted Approach 4. This is more computationally efficient than 3, and requires much less data than 1 and 2. However, it has the potential to introduce some artifacts, which come from seeding infection into the population in patterns that are unlikely to have emerged naturally. For example, a natural population would see some level of correlation in the HIV status of main partners, and that correlation would be stronger the longer the two partners were together; the exact pattern would depend on the HIV transmission dynamics that are consistent with all of the model features in combination. To avoid including in our outcome measures the transient dynamics that occur while our initially seeded population is achieving a more internally consistent state, we simulated a burn-in period and only then began measuring the results for our baseline model.

In the end, we were highly conservative, and used a 100-year burn-in. However, a subsequent analysis of the burn-in suggests that a far shorter period would have been acceptable, at least in terms of our core metrics of transmissions attributable to various categories. Figure S1 provides an example, showing the proportion of transmissions occurring within various categories by year over the course of the burn-in for Peru Model 1. Some categories are excluded for readability; all of these can all be calculated from those shown (e.g. transmissions from casual partners = 1 – those from main partners; transmissions in AIDS stage are 1 – those in acute stage – those in chronic stage). We see that these metrics do indeed show some burn-in effect, but they settle into their equilibrium distributions within about 20 years, far shorter than the 100 years that we included, and less than the time the HIV epidemic has unfolded within these populations. The three other cases (US Model 1, US Model 2, Peru Model 2) are all qualitatively similar, and available from the corresponding author upon request. HIV incidence also takes about 20-30 years to settle into an equilibrium; prevalence, of course, as a “lagging indicator”, takes longer (Figure S2)**.**

It is important to reiterate that this burn-in period does not represent past calendar time (i.e. we are not using Approach 1 or 2). It merely serves to ensure that our initial populations are internally consistent, while reflecting recent behavioral, testing and treatment data to the largest extent possible.

**Model replication and stochastic variation**

Each model was run 10 times, to document stochastic variation and allow for interpretation of differences across countries and models. We stopped at 10 runs, given the intense computational burden (~8-10 days/run on a UNIX cluster) and the very small stochastic variation observed for key outcome measures. This small variation was driven to a large extent by the fact that we were averaging our outcome measures across the entire 25 year simulation period. As discussed in the main text, considering smaller intervals is of interest as well. We present year-on-year variation in outcome measures for the US Model 2 there, and include the remaining three country/model combinations here, in Figure S3.

**FIGURE S1: Outcome measures by year throughout burn-in, Peru Model 1**

**
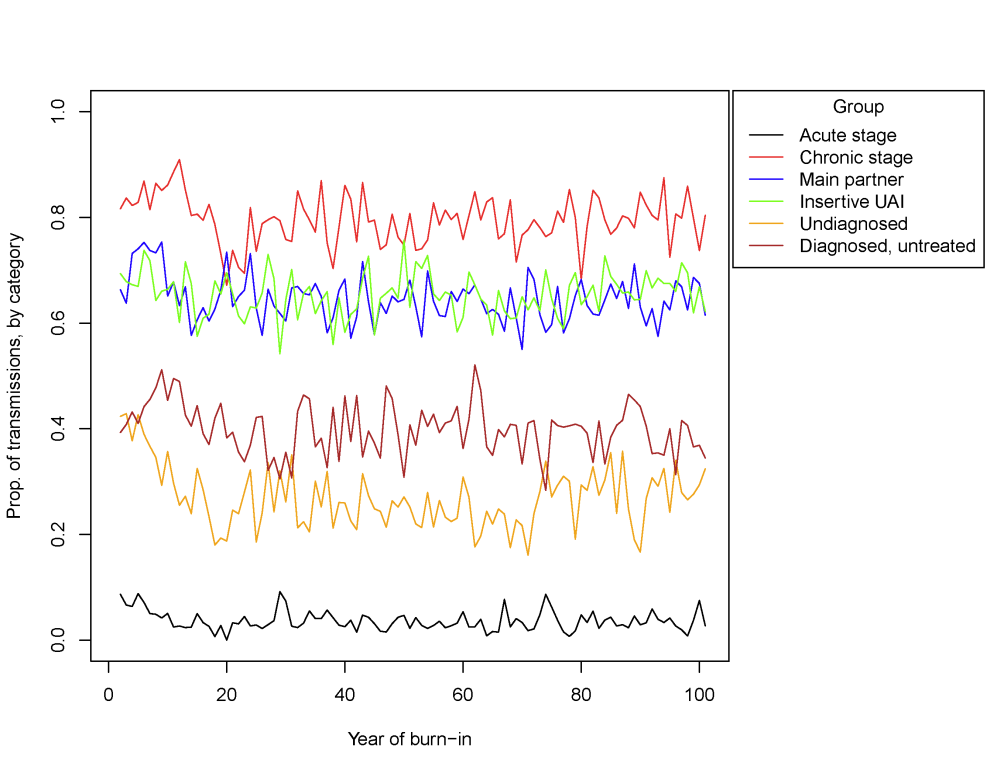
**

**FIGURE S2**

**
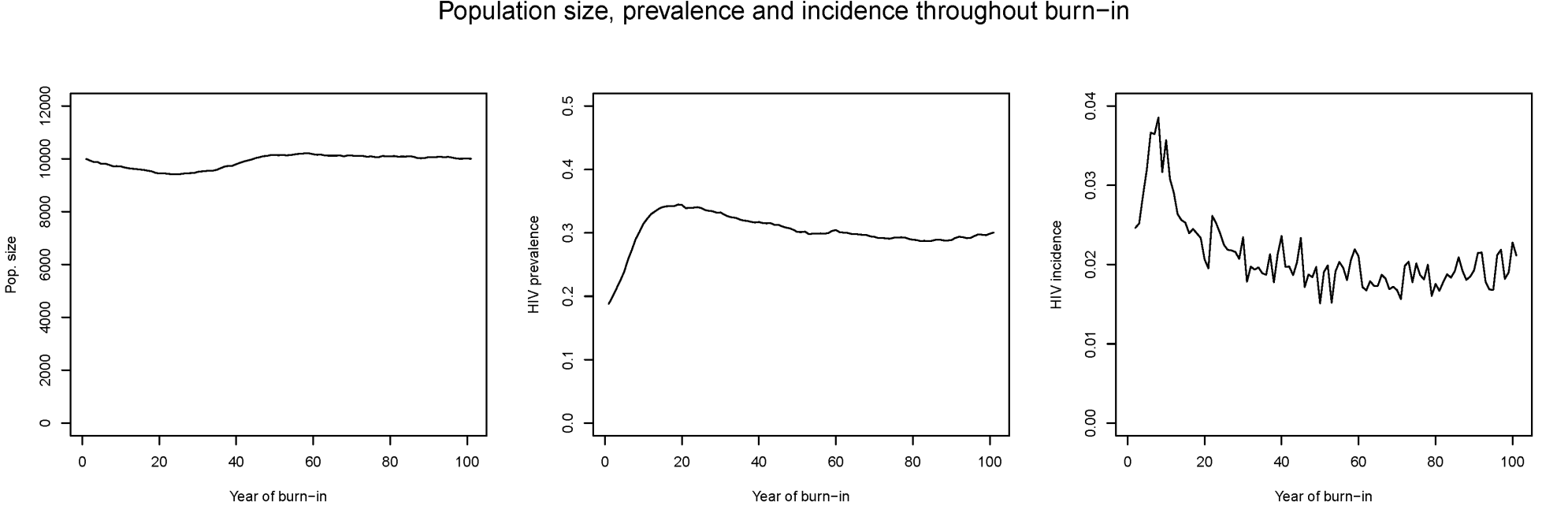
**

**FIGURE S3: Proportion of transmission by category – variation across single years**

See main text for US Model 2.

**
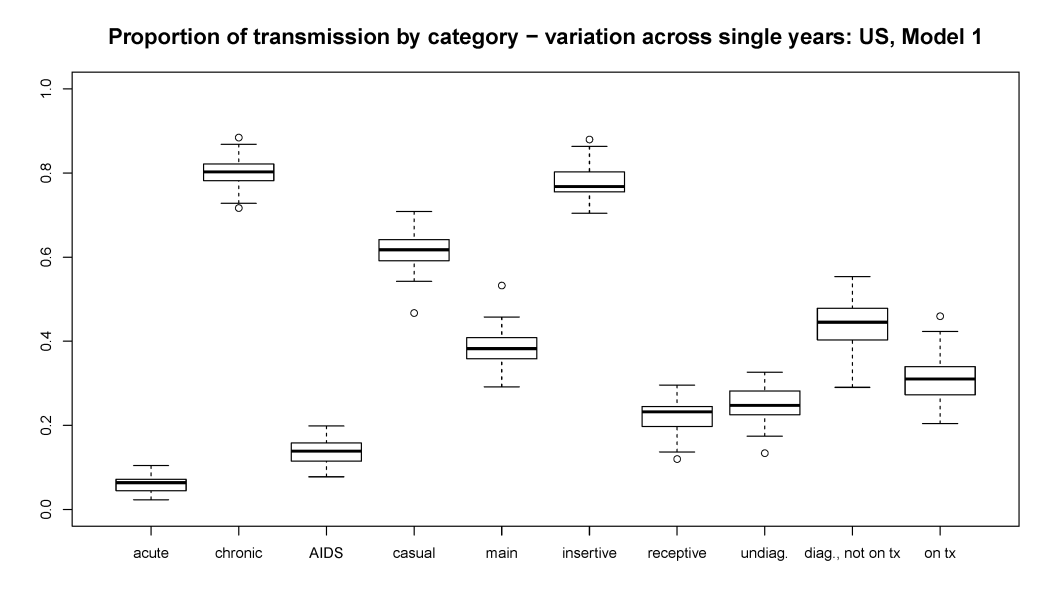

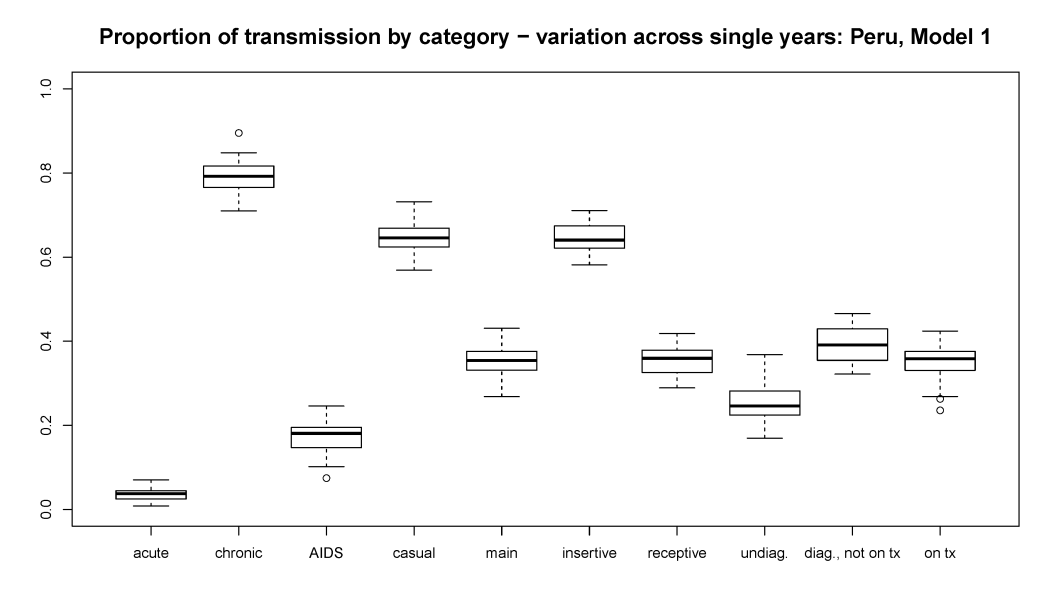

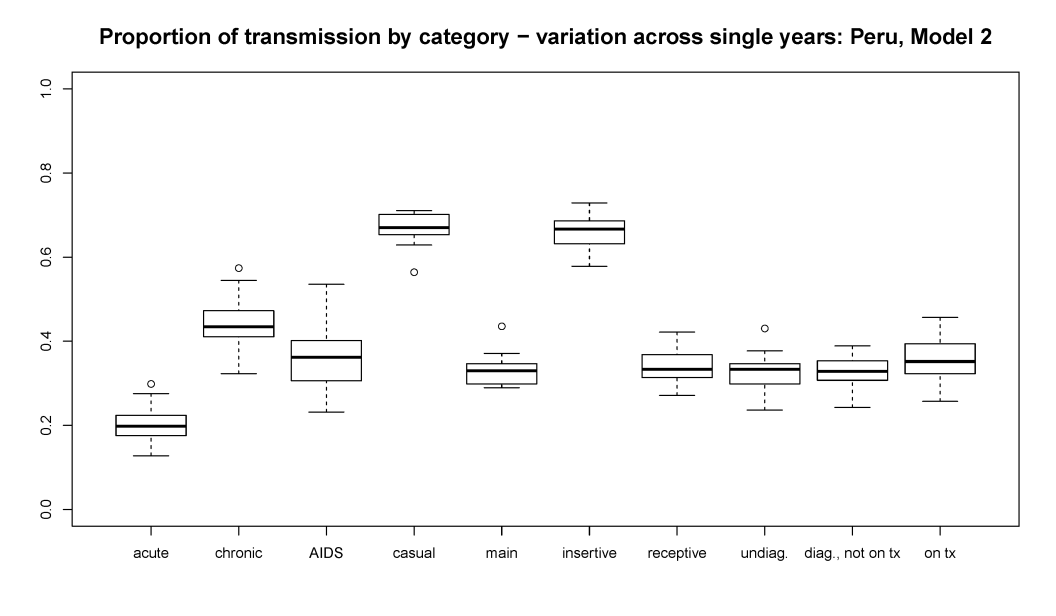
**

**References**

1. Chesney MA, Koblin BA, Barresi PJ, Husnik MJ, Celum CL, et al. (2003) An individually tailored intervention for HIV prevention: baseline data from the EXPLORE Study. Am J Public Health 93: 933-938.

2. Koblin BA, Chesney MA, Husnik MJ, Bozeman S, Celum CL, et al. (2003) High-risk behaviors among men who have sex with men in 6 US cities: baseline data from the EXPLORE Study. Am J Public Health 93: 926-932.

3. Goicochea P, Sanchez J, Lucchetti A, Kusunoki L, Galvan R, et al. (2004) Successful recruitment and retention of high-risk men who have sex with men (MSM) in a preparedness cohort (HPTN 036) for an HSV-2 suppression trial: high observed incidence of HIV-1 infection. XV International AIDS Conference. Bangkok: International AIDS Society.

4. Celum C, Wald A, Hughes J, Sanchez J, Reid S, et al. (2008) Effect of aciclovir on HIV-1 acquisition in herpes simplex virus 2 seropositive women and men who have sex with men: a randomised, double-blind, placebo-controlled trial. Lancet 371: 2109-2119.

5. (2010) Prevalence and awareness of HIV infection among men who have sex with men --- 21 cities, United States, 2008. MMWR Morb Mortal Wkly Rep 59: 1201-1207.

6. Sanchez T, Finlayson T, Drake A, Behel S, Cribbin M, et al. (2006) Human immunodeficiency virus (HIV) risk, prevention, and testing behaviors--United States, National HIV Behavioral Surveillance System: men who have sex with men, November 2003-April 2005. MMWR Surveill Summ 55: 1-16.

7. MacKellar DA, Gallagher KM, Finlayson T, Sanchez T, Lansky A, et al. (2007) Surveillance of HIV risk and prevention behaviors of men who have sex with men - A national application of venue-based, time-space sampling. Public Health Reports 122: 39-47.

8. Lama JR, Sanchez J, Suarez L, Caballero P, Laguna A, et al. (2006) Linking HIV and antiretroviral drug resistance surveillance in Peru: a model for a third-generation HIV sentinel surveillance. J Acquir Immune Defic Syndr 42: 501-505.

9. Grohskopf L, Gvetadze R, Pathak S, O'Hara B, Mayer K, et al. (2010) Preliminary analysis of biomedical data from the phase II clinical safety trial of tenofovir disoproxil fumarate (TDF) for HIV-1 pre-exposure prophylaxis (PrEP) among U.S. men who have sex with men (MSM). XVIII International AIDS Conference. Vienna: International AIDS Society.

10. McElrath MJ, De Rosa SC, Moodie Z, Dubey S, Kierstead L, et al. (2008) HIV-1 vaccine-induced immunity in the test-of-concept Step Study: a case-cohort analysis. Lancet 372: 1894-1905.

11. Buchbinder SP, Mehrotra DV, Duerr A, Fitzgerald DW, Mogg R, et al. (2008) Efficacy assessment of a cell-mediated immunity HIV-1 vaccine (the Step Study): a double-blind, randomised, placebo-controlled, test-of-concept trial. Lancet 372: 1881-1893.

12. Moorman AC, Holmberg SD, Fuhrer J, Loveless M, Von Bargen J, et al. (1996) The HIV Outpatient Study (HOPS): description and initial findings. International Conference on AIDS. Vancouver. pp. 100.

13. Arias E (2007) United States Life Tables, 2004. Atlanta: US Centers for Disease Control and Prevention, Division of Vital Statistics.

14. Heron M, Tejada-Vera B (2009) Deaths: Leading Causes for 2005. Atlanta: US Centers for Disease Control and Prevention, Division of Vital Statistics.

15. World Health Organization (2008) Life table: Peru, Male.

16. Helms DJ, Weinstock HS, Mahle KC, Bernstein KT, Furness BW, et al. (2009) HIV testing frequency among men who have sex with men attending sexually transmitted disease clinics: implications for HIV prevention and surveillance. J Acquir Immune Defic Syndr 50: 320-326.

17. Swindells S, Cobos DG, Lee N, Lien EA, Fitzgerald AP, et al. (2002) Racial/ethnic differences in CD4 T cell count and viral load at presentation for medical care and in follow-up after HIV-1 infection. AIDS 16: 1832-1834.

18. Lyles RH, Munoz A, Yamashita TE, Bazmi H, Detels R, et al. (2000) Natural history of human immunodeficiency virus type 1 viremia after seroconversion and proximal to AIDS in a large cohort of homosexual men. Multicenter AIDS Cohort Study. J Infect Dis 181: 872-880.

19. Moreira RI, Luz PM, Struchiner CJ, Morgado M, Veloso VG, et al. (2011) Immune Status at Presentation for HIV Clinical Care in Rio de Janeiro and Baltimore. Jaids-Journal of Acquired Immune Deficiency Syndromes 57: S171-S178.

20. Chu H, Gange SJ, Li X, Hoover DR, Liu C, et al. (2010) The effect of HAART on HIV RNA trajectory among treatment-naive men and women: a segmental Bernoulli/lognormal random effects model with left censoring. Epidemiology 21 Suppl 4: S25-34.

21. Weintrob AC, Grandits GA, Agan BK, Ganesan A, Landrum ML, et al. (2009) Virologic response differences between African Americans and European Americans initiating highly active antiretroviral therapy with equal access to care. J Acquir Immune Defic Syndr 52: 574-580.

22. Ministry of Health of Peru (2006) A step forward in the fight against AIDS: the first two years of universal access to antiretroviral treatment in Peru. Lima: Ministry of Health.

23. Wawer MJ, Gray RH, Sewankambo NK, Serwadda D, Li X, et al. (2005) Rates of HIV-1 transmission per coital act, by stage of HIV-1 infection, in Rakai, Uganda. J Infect Dis 191: 1403-1409.

24. Little SJ, McLean AR, Spina CA, Richman DD, Havlir DV (1999) Viral dynamics of acute HIV-1 infection. J Exp Med 190: 841-850.

25. Leynaert B, Downs AM, de Vincenzi I (1998) Heterosexual transmission of human immunodeficiency virus: variability of infectivity throughout the course of infection. European Study Group on Heterosexual Transmission of HIV. Am J Epidemiol 148: 88-96.

26. Buchbinder SP, Katz MH, Hessol NA, O'Malley PM, Holmberg SD (1994) Long-term HIV-1 infection without immunologic progression. AIDS 8: 1123-1128.

27. Robins G, Morris M (2007) Advances in exponential random graph (p*) models. Social Networks 29: 169-172.

28. Wasserman S, Pattison P (1996) Logit models and logistic regressions for social networks: I. an introduction to Markov graphs and p*. Psychometrika 60: 401-425.

29. Snjiders TAB (2002) Markov Chain Monte Carlo Estimation of Exponential Random Graph Models. Journal of Social Structure 3: 1-40.

30. Carnegie N, Hunter D, Goodreau S (2011) Cross-Sectional Approximations to Separable Temporal ERGM Parameters. Sunbelt XIII: International Network for Social Network Analysis. St. Pete Beach, FL.

31. Butts CT (2008) network: a Package for Managing Relational Data in R. Journal of Statistical Software 24.

32. Butts CT, Handcock MS, Hunter DR (2011) network: Classes for Relational Data. 1.6 ed. Irvine, Calif.

33. Krivitsky PN, Handcock MS, Morris M (2011) Adjusting for network size and composition effects in exponential-family random graph models. Statistical Methodology 8: 319-339.

34. Wood E, Montaner JS, Tyndall MW, Schechter MT, O'Shaughnessy MV, et al. (2003) Prevalence and correlates of untreated human immunodeficiency virus type 1 infection among persons who have died in the era of modern antiretroviral therapy. J Infect Dis 188: 1164-1170.

35. Vittinghoff E, Douglas J, Judson F, McKirnan D, MacQueen K, et al. (1999) Per-contact risk of human immunodeficiency virus transmission between male sexual partners. Am J Epidemiol 150: 306-311.

36. Gray RH, Kigozi G, Serwadda D, Makumbi F, Watya S, et al. (2007) Male circumcision for HIV prevention in men in Rakai, Uganda: a randomised trial. Lancet 369: 657-666.

37. Wilson DP, Law MG, Grulich AE, Cooper DA, Kaldor JM (2008) Relation between HIV viral load and infectiousness: a model-based analysis (vol 372, pg 314, 2008). Lancet 372: 1808-1808.

38. (2005) HIV prevalence, unrecognized infection, and HIV testing among men who have sex with men--five U.S. cities, June 2004-April 2005. MMWR Morb Mortal Wkly Rep 54: 597-601.
